# Functional iridoid synthases from iridoid producing and non-producing *Nepeta* species (subfam. Nepetoidae, fam. Lamiaceae)

**DOI:** 10.3389/fpls.2023.1211453

**Published:** 2024-01-03

**Authors:** Neda Aničić, Dragana Matekalo, Marijana Skorić, Uroš Gašić, Jasmina Nestorović Živković, Slavica Dmitrović, Jelena Božunović, Milica Milutinović, Luka Petrović, Milena Dimitrijević, Boban Anđelković, Danijela Mišić

**Affiliations:** ^1^ Institute for Biological Research “Siniša Stanković” - National Institute of the Republic of Serbia, University of Belgrade, Belgrade, Serbia; ^2^ Institute for Multidisciplinary Research, University of Belgrade, Belgrade, Serbia; ^3^ Faculty of Chemistry, University of Belgrade, Belgrade, Serbia

**Keywords:** iridoid synthase, iridoids, *Nepeta rtanjensis*, *Nepeta nervosa*, functional characterization, metabolomics, transcriptomics

## Abstract

Iridoids, a class of atypical monoterpenes, exhibit exceptional diversity within the *Nepeta* genus (subfam. Nepetoidae, fam. Lamiaceae).The majority of these plants produce iridoids of the unique stereochemistry, with nepetalactones (NLs) predominating; however, a few *Nepeta* species lack these compounds. By comparatively analyzing metabolomics, transcriptomics, gene co-expression, and phylogenetic data of the iridoid-producing *N. rtanjensis* Diklić & Milojević and iridoid-lacking *N. nervosa* Royle & Bentham, we presumed that one of the factors responsible for the absence of these compounds in *N. nervosa* is iridoid synthase (ISY). Two orthologues of ISY were mined from leaves transcriptome of *N. rtanjensis* (*Nr*PRISE1 and *Nr*PRISE2), while in *N. nervosa* only one (*Nn*PRISE) was identified, and it was phylogenetically closer to the representatives of the Family 1 isoforms, designated as P5βRs. Organ-specific and MeJA-elicited profiling of iridoid content and co-expression analysis of IBG candidates, highlighted *NrPRISE2* and *NnPRISE* as promising candidates for ISY orthologues, and their function was confirmed using *in vitro* assays with recombinant proteins, after heterologous expression of recombinant proteins in *E. coli* and their His-tag affinity purification. *Nr*PRISE2 demonstrated ISY activity both *in vitro* and likely *in planta*, which was supported by the 3D modeling and molecular docking analysis, thus reclassification of *Nr*PRISE2 to *Nr*ISY is accordingly recommended. *Nn*PRISE also displays *in vitro* ISY-like activity, while its role under *in vivo* conditions was not here unambiguously confirmed. Most probably under *in vivo* conditions the *Nn*PRISE lacks substrates to act upon, as a result of the loss of function of some of the upstream enzymes of the iridoid pathway. Our ongoing work is conducted towards re-establishing the biosynthesis of iridoids in *N. nervosa*.

## Introduction

Nepetoidae subfamilly of the Lamiaceae family comprises mainly iridoid-lacking taxa. It has been proposed that, during evolution, these plants have lost a key enzyme in the early iridoid pathway, iridoid synthase (ISY) (Boachon et al., 2018), which disabled their iridoid biosynthetic platform. The exception are members of the genus *Nepeta*, which went through the re-establishment of the iridoid biosynthesis by engaging the latent biosynthetic machinery existing in all Nepetoidae, in parallel with convergent evolution of ISY from an alternative ancestor, progesterone 5β-reductase (P5βR) (Lichman et al., 2020). Another evolutionary innovation of the genus *Nepeta* includes NAD-dependent nepetalactol-related short-chain-dehydrogenase/reductase (NEPS) and major latex protein-like (MLPL) enzymes, which, in combination with novel ISYs, gave rise to iridoid aglycones nepetalactones and glycosylated iridoids of unique stereochemistry, exclusively present in this group of plants.

**Figure 1:**
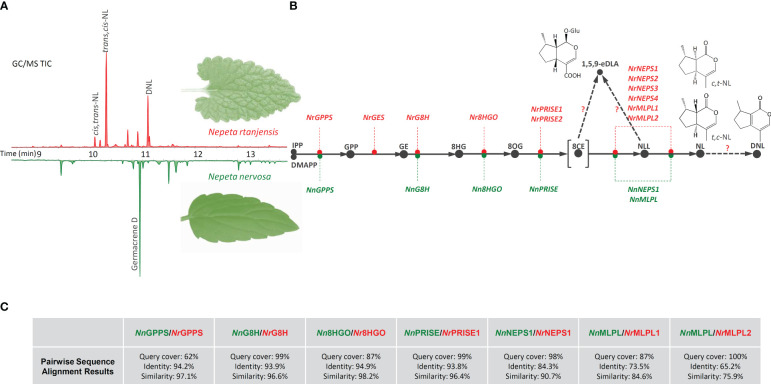
Following GC/MS and UHPLC/QToF MS characterization of methanol extracts of *N. rtanjensis* and *N. nervosa* leaves, iridoid compounds were identifyed only in *N. rtanjensis*, with *t*,*c*-NL, *c*,*t*-NL, and DNL predominating on the **(A)** representative GC/MS chromatogram. In parallel, orthologues of iriroid biosynthetic genes were searched in transcriptomes of *N. rtanjensis* (red color) and *N. nervosa* (green color) and the biosynthetic pathway of the two species was reconstructed **(B)**: GPPS- geranyl diphosphate synthase, GES- geraniol synthase, G8H- geraniol 8-hydroxylase, 8HGO- 8-hydroxygeraniol oxidoreductase, PRISE- progesterone-5β-reductase/iridoid synthase activity displaying enzymes, NEPS- nepetalactol-related short-chain dehydrogenase, MLPL- major latex protein-like enzyme. Pairwise sequence alignment results for amino acid sequences of investigated genes belonging to *N. nervosa* with the corresponding *N. rtanjensis* genes **(C)**. IPP, isopentenyl pyrophosphate; DMAPP, dimethylallyl pirophosphate; GPP, geranyl pyrophosphate; GE, geraniol; 8HG, 8-hydroxygeraniol; 8OG, 8-oxogeranial; 8CE – 8-oxocitronellyl enolate; NLL, nepetalactol; NL, nepetalactone; DNL, 5,9-dehydronepetalactone; 1,5,9-*e*DLA, 1,5,9-*epi*deoxyloganic acid; *t*,*c*-NL, *trans*,*cis*-nepetalactone, *c*,*t*-NL, *cis*,*trans*-nepetalactone.

All iridoids in *Nepeta* have a common precursor nepetalactol, which emerges from geranyl pyrophosphate (GPP) originating from the MEP pathway. This central precursor arises in a reaction assisted by the GPP synthase (GPPS), and is further converted into geraniol via geraniol synthase (GES). Nepetalactol arises from geraniol through the series of intermediates and enzymatic reactions catalyzed by geraniol 8-hydroxylase (G8H), 8-hydroxygeraniol oxidoreductase (8HGO), ISY, NEPS(s), and MLPL (**Figure 1**). In *Nepeta* species, ISYs are mainly responsible for the stereoselective 1,4-reduction of 8-oxogeranial to uncyclized and reactive 8-oxocitronellyl enol, and for determining the stereochemistry of the C7 (Sherden et al., 2017; Lichman et al., 2019b; Lichman et al., 2020; Hernández Lozada et al., 2022). In other iridoid producing plants, including *Catharanthus roseus* L. (Geu-Flores et al., 2012) and *Olea europea* (Alagna et al., 2016) ISYs are responsible for reduction of 8-oxogeranial. The subsequent cyclization step, which gives rise to a core iridoid skeleton characteristic for nepetalactol, is in these plants either mediated by some unknown cyclases, or it occurs spontaneously. Enolate intermediate 8-oxocitronellyl enol in *Nepeta* is cyclized by NEPSs (Lichman et al., 2019a; Lichman et al., 2019b), but can also undergo a spontaneous cyclization to produce predominately *cis*,*trans*- steroisomer of nepetalactol (Hernández Lozada et al., 2022). NEPS enzymes are also involved in the subsequent enzymatic step, which converts nepetalactol to nepetalactone, and are responsible for setting the stereochemistry of the bridged carbons (C4a and C7a) of nepetalactone (Hernández Lozada et al., 2022). In summary, the family of NEPS enzymes can be divided into 3 subgroups according to their catalytic activity: 1) redox-inactive cyclases (e.g. *N. mussinii Nm*NEPS3); 2) oxidazes (e.g. *Nm*NEPS1 and *Nm*NEPS5); 3) dual-function enzymes catalyzing both stereo selective cyclization and oxidation of various nepetalactols to nepetalactons (e.g. *N. cataria Nc*NEPS3A; *N. mussinii Nm*NEPS4; *N. sibirica Ns*NEPS2) (Lichman et al., 2020; Hernández Lozada et al., 2022). MLPLs are proven to be involved in the biosynthesis of *cis,trans*-nepetalactol stereoisomer (Lichman et al., 2020).

The story of iridoid biosynthesis within the genus *Nepeta* becomes even more fascinating when it comes to taxa lacking iridoids, or producing them in trace amounts. We here hypothesized that a “biochemical reservoir” of enzymes with different catalytic activities related to the iridoid biosynthesis exists in iridoid non-producing *Nepeta* species, but substrates to act upon are missing due to the loss of function/silencing of some of the early biosynthetic genes. In order to test this hypothesis we focused our study towards two chemodiverse *Nepeta* taxa: 1) *N. rtanjensis* Diklić & Milojević, an endemic and critically endangered plant of Serbia, which is characterized by the presence of nepetalactones with *trans,cis*- and *cis,trans*- stereochemistry; and 2) iridoid-lacking *N. nervosa* Royle ex Benth. Following comprehensive metabolomics, we analyzed transcriptomes of *N. rtanjensis* and *N. nervosa* leaves in search for iridoid-related biosynthetic genes, to acquire accurate information on the presence/absence of iridoid biosynthetic gene transcripts and gene nucleotide sequence. Candidates of iridoid biosynthetic genes (*GPPS*, *GES*, *G8H*, *HGO*, *ISY*, *NEPSs*, *and MLPLs*) are identified based on similarity to the previously characterized orthologues from *Nepeta cataria*, *N. mussinii*, *N. rtanjensis*, *N. sibirica*, *C. roseus*, and other iridoid-rich species. As ISYs were identified as genes responsible for the loss of iridoid biosynthesis in the majority of Nepetoidae, and are highlighted as enzymes important for determining the metabolic flux through the pathway, we further aimed to isolate and functionally characterize ISYs from *N. rtanjensis* and *N. nervosa*, and analyze them in a phylogenetic context. Furthermore, it has recently been suggested that the presence or absence of ISY-like enzymatic activity controls whether plants accumulate nepetalactones, or whether these molecules are of 7*S* or 7*R* configuration (Hernández Lozada et al., 2022). Iridoids with *7R* stereochemistry predominate outside of *Nepeta* genus, while, up to date, only 7*S* isomers of iridoids are recorded within *Nepeta*, which coincides with the fact that only 7*S*-specific ISYs from *N. cataria* and *N. mussinii* (Sherden et al., 2017), and *N. sibirica* (Hernández Lozada et al., 2022) have been isolated and functionally characterized. As transcripts of only one potential PRISE were recorded in transcriptome of *N. nervosa*, we aimed to investigate whether the loss of function or silencing of this important IBG is, at least partially, responsible for the lack of iridoids in this species.

## Materials and methods

### Plant material and *in vitro* culture establishment

Seeds of *N. nervosa* were commercially purchased from Grugapark Essen (Germany), while seeds of *N. rtanjensis* were collected in July 2017 in locality Javor (Mt Rtanj, SE Serbia). Seeds were surface sterilized in a 20% solution of commercial bleach for 10 min, rinsed five times with sterile distilled water and germinated in Petri dishes on 20 ml basal medium (BM): solid ½ MS (Murashige and Skoog, 1962) culture medium, supplemented with 20 g l^-1^ sucrose, 7 g l^-1^ agar (Torlak, Serbia) and 100 mg l^-1^ myo-inositol (Merck, Germany). All the cultures were grown in 370 ml glass jars, each containing 100 ml of BM, and kept in a growth chamber under long day conditions (16/8 h light/dark cycle), at 25 ± 2°C. White fluorescent tubes provided a photon flux rate of 32.5 μmol m^2^ s^-1^ at the level of plant cultures.

Sufficient plant material for experiments was obtained by micropropagation of one selected genotype of each of the *Nepeta* species, using single-node stem segments as explants, and by subcultivation on fresh BM every 4 weeks. RNA-seq libraries were constructed using leaves of four-week old *in vitro* grown *N. rtanjensis* and *N. nervosa* plants. Leaves, stems, and roots of four month old plants were separately harvested, weighted and immediately frozen in liquid nitrogen, and further stored at −80°C until use. Three individuals (three biological replicates) of one clonally propagated genotype per species, at the same developmental stage, were analyzed separately.

### MeJA-elicitation experimental setup

Four weeks-old *N. rtanjensis* and *N. nervosa* plants, propagated on BM under *in vitro* conditions, were used in experiments to comparatively analyze methyl jasmonate (MeJA)-elicitation effects on iridoid profile and related BG expressions. Plants were transferred on BS supplemented with 250 μM MeJA and leaves were harvested after 24 h and 72 h. Filter-sterilized MeJA (sterile 0.2 μm cellulose filters, Agilent Technologies, Santa Clara, CA, USA) was added to the culture medium after its sterilization by autoclaving at 114°C for 25 min. Control group of plants was simultaneously transferred on BM, and leaves were harvested in parallel with those of MeJA-treated plants. Each of the three biological replicates per treatment consisted of five clonally propagated plants grown in the same jar. After harvesting, leaves belonging to the plants of the same biological replicate were pulled, ground into a fine powder and homogenized in liquid nitrogen, and samples were stored at −80°C until use. The obtained plant material was used for both RNA extraction and quantification of iridoids.

### Extraction of plant material for metabolic profiling

Plant material was ground in LN, and extracted with 96% methanol (w:v = 1:10) in an ultrasonic bath for 1 h. After centrifugation for 20 min at 10000 × g, the supernatants were filtered through 0.2μm cellulose filters (Agilent Technologies, Santa Clara, CA, USA) and stored at 4°C until use. All the analyses were performed in triplicates.

### GC/MS non-targeted metabolomics of methanol extracts of *N. rtanjensis* and *N. nervosa* leaves

Profiling of volatile compounds in leaves of *N. rtanjensis* and *N. nervosa* grown *in vitro* was performed using Agilent 8890 gas chromatography (GC) System with 5977B GC/MSD (Agilent Technologies, USA) connected to Centri sample extraction and enrichment platform (Markes International Ltd., UK). Chromatographic separations were performed on HP-5MS column (30 m × 0.25 mm, 0.25 μm film thickness) (Agilent Technologies, USA), and using He (99.999%, The Linde Group, Ireland) as a carrier gas at a flow rate of 1.6 ml min^−1^. Transfer line was heated at 280°C, and detector temperature was set to 270°C. Mass spectra were acquired in positive EI mode (+70 eV), with temperature of the EI source set to 280°C. Column temperature was linearly programmed from 40 to 300°C, at rate of 20°C min^−1^, and held isothermally at 240°C for the next 10 min. Methanol extract (1 μl) was injected in a split mode (20:1), with split flow 24 ml min^-1^. Analyses were performed in SCAN mode, tracking the compounds within the range 45 to 500 amu. The constituents of the reaction mixtures were identified by comparison of their mass spectra and retention times with those of the respective standards, and by comparison with the NIST05 library.

### UHPLC−QToF−MS non-targeted metabolomics of *N. rtanjensis* and *N. nervosa* methanol extracts

The analyses were carried out on Agilent 1290 Infinity ultra-high-performance liquid chromatography (UHPLC) system coupled with a quadrupole time-of-flight mass spectrometry (6530C Q-ToF-MS) from Agilent Technologies, Inc., CA, USA. The chromatographic separation was performed at 40°C on a Zorbax C18 column (2.1 × 50 mm, 1.8 µm) from Agilent Technologies, Inc., CA, USA. The composition of mobile phases, gradient elution program, and the all chromatography parameters were as previously described by Gašić et al., 2023.

The QToF-MS system was equipped with an Agilent Jet Stream electrospray ionization (ESI) source, operating in both positive (ESI+) and negative (ESI-) ionization modes. The operation parameters for ESI, as well as the other settings of the QToF mass analyzer and data-dependent acquisition (DDA) parameters were the same as in Kostić et al., 2023. Agilent MassHunter software was used for data acquisition. The CAS SciFindern database was used to search for chemical compounds by formulas and structures (https://scifinder-n.cas.org/). For the evaluation of MS data R Studio software (enviPick and xcms R packages) was used (Zengin et al., 2020).

### UHPLC/(+)HESI-MS/MS quantification of targeted iridoids

Dionex Ultimate 3000 UHPLC system (ThermoFisher Scientific, Germany) connected to TSQ Quantum Access Max triple-quadrupole mass spectrometer (ThermoFisher Scientific, Switzerland) was used for the determination and quantification of nepetalactone, dehydronepetalactone, and 1,5,9-*epi*deoxyloganic acid in methanol extracts of *N. rtanjensis* and *N. nervosa* leaves, stems and roots, as well as in MeJA-elicited leaves. Elution was performed at 40°C on Hypersil gold C18 column (50 × 2.1 mm) with 1.9 m particle size (ThermoFisher Scientific, USA). The liquid chromatography parameters (mobile phase, gradient elution, and the flow rate) and the mass spectrometry detection settings were set according to Mišić et al., 2015. Mass spectrometry data were acquired in both positive (*trans*,*cis*-nepetalactone, *cis*,*trans*-nepetalactone, and dehydronepetalactone) and negative (1,5,9-*epi*deoxyloganic acid) mode, and collision-induced fragmentations were performed using argon, with collision energy (cE) set to 30 eV. The identification of targeted compounds was additionally confirmed by DAD analysis. Absorption spectrum of nepetalactone isomers was characterized by the λ_max_ at 230 nm, while dehydronepetalactone had a max absorption at λ_max_ = 300 nm. As for 1,5,9-*epi*deoxyloganic acid, its absorption spectra was characterized by λ_max_ = 340 nm.

The amounts of *trans,cis-*nepetalactone, dehydronepetalactone, and 1,5,9*-epi*deoxy*l*oganic acid were evaluated by calculating the peak areas, based on the calibration curve of pure compounds, as previously described in Aničić et al., 2021. Amounts were expressed as µg per 100 mg of fresh weight (µg 100 mg^-1^ FW). All the analyses were performed in triplicates.

### Transcriptome mining and selection of iridoid biosynthesis candidate genes

Transcriptomes of nepetalactone-producing *Nepeta rtanjensis* Diklić & Milojević and nepetalactone-lacking *N. nervosa L.* are searched for the presence/absence of transcripts of iridoid-pathway-related genes (biosynthetic genes and transcription factors), based on the homology with the genes characterised in many iridoids- and alkaloids- producing plants, including those from the *Nepeta* genus (Lichman et al., 2019a; Lichman et al., 2020; Hernández Lozada et al., 2022). Sequences of *N. rtanjensis* iridoid-biosynthetic-pathway-genes coding for *Nr*GPPS.SSU, *Nr*GES, *Nr*G8H, *Nr*8HGO, *Nr*PRISE1, *Nr*PRISE2, *Nr*MLPL1, *Nr*MLPL2, *Nr*NEPS1, *Nr*NEPS2, *Nr*NEPS3, and *Nr*NEPS4 (Aničić et al., 2020) were used for BLAST search of corresponding candidate genes in *N. nervosa* leaf RNA-seq available in our laboratory (data not published). BLAST search derived 6 genes putatively involved in iridoid biosynthesis – *NnGPPS.SSU*, *NnG8H*, *Nn8HGO*, *NnMLPL*, *NnPRISE*, and *NnNEPS1* from *N. nervosa* transcriptomic database. All of the sequences have shown high similarity to the characterized genes from other species present in the NCBI database. For the NCBI accession numbers of all genes from this paper please refer to the **Supplementary Table 1**.

### RNA extraction and qPCR profiling of tissue-specific and MeJA-elicited expression of iridoid-related biosynthetic genes

Once the candidates for the iridoid biosynthesis-related genes were identified in *N. rtanjensis* and *N. nervosa* transcriptomes, highly specific primer pairs for qPCR co-expression analysis were designed using Primer3Plus software (http://www.bioinformatics.nl/cgi-bin/primer3plus/primer3plus.cgi) (**Supplementary Table 1**). Finally, glyceraldehyde 3-phosphate dehydrogenase (GAPDH) was used as the housekeeping gene, as previously reported by Aničić et al., 2018.

The RNA extraction from leaves, stems, and roots of *N. rtanjensis* and *N. nervosa*, as well as from MeJA-elicited leaves, was performed applying a modified CTAB method (Gasic et al., 2004). RNA was quantified with N60 Nano-Photometer® (Implen GmbH, Munich, Germany) and fluorometrically (Qubit 3.0 Fluorometer, ThermoFisher Scientific, USA), and its integrity was confirmed with gel electrophoresis. Obtained total RNA was treated with DNaze I (ThermoFisher Scientific, USA) for 30 min at 37°C. From 1 μg of total RNA cDNA was constructed using the Revert Aid First Strand cDNA Synthesis Kit (ThermoFisher Scientific, USA) following manufacturers’ specifications, with oligo-(dT) primers (Life Technologies, USA). The PCR mixture comprised of cDNA corresponding to 50 ng of total RNA, 1µM primers, 1U of Phusion Hot Start II High-Fidelity polymerase (Thermo Scientific, USA) in a volume of 50 µl. The amplification was carried out in an Eppendorf Mastercycler Nexus (Eppendorf, Germany) thermal cycler with the following amplification profile: initial denaturation (30 s at 98°C), followed by 40 cycles of denaturation (10 s at 98°C), annealing (30 s at 60°C), and extension (45 s at 72°C) with final extension (7 min at 72°C). The obtained amplicons were purified electrophoretically, extracted from gel using GeneJET Gel extraction kit (Thermo Scientific, USA), quantified and serially diluted in a 10^9^-10^2^ copies µl^-1^ range to be used as standards for the absolute qPCR quantification.

Gene expression analyses were performed by real-time PCR using QuantStudio™ 3 Real-Time PCR System (Life Technologies, USA). Thermocycler conditions were as previously described in Aničić et al., 2018. The reactions were performed using Maxima SYBR Green/ROX Master Mix (2X) (ThermoFisher Scientific, USA), cDNA corresponding to 50 ng RNA and 0.3 µM primers, according to the manufacturer’s recommendations. The expression levels of candidate IBGs were calculated according to the 2^−ΔΔCt^ method (Livak and Schmittgen, 2001) using GAPDH as a housekeeping gene, as mentioned above. The data represent means ± SE from three biological replicates.

### *Nr*PRISE2 and *Nn*PRISE amplification and cloning

Primers for the full-length amplification of candidate ISYs are presented in **Supplementary Table 1**. Total *N. rtanjensis* and *N. nervosa* leaf RNAs were isolated with Spectrum™ Plant Total RNA kit (Sigma-Aldrich®, Hamburg, Germany), and cDNA was synthesized using RevertAid First Strand cDNA Synthesis kit (ThermoScientific, Lithuania) following manufacturers’ instructions. Full lengths of the selected genes were amplified using AmpliTaq Gold (Thermo Fisher Scientific, USA) and cDNAs as templates. PCR products were gel purified and the products were subsequently cloned into vector PTZ57R/T using InsTAclone PCR Cloning Kit (ThermoScientific, Lithuania). The obtained constructs were used for PCR amplification of candidate PRISEs genes with primers containing restriction enzymes sites (KpnI and SacI) (**Supplementary Table 1**). After digestion with the appropriate restriction enzymes the digests were ligated to bacterial expression vector pRSETA (N-terminal 6xHis tag). Final constructs were verified by sequencing.

### *Nr*PRISE2 and *Nn*PRISE expression in bacteria and protein purification

The pRSETA-NrPRISE2 and pRSETA-NnPRISE constructs were used for heat-shock transformation of *E. coli* strain BL21-CodonPlus (DE3)-RIL (Stratagene, USA). Single colonies were grown in Lauria-Bertani (LB) broth supplemented with 100 μg ml^-1^ ampicillin, 50 μg ml^-1^ kanamycin and 17 μg ml^-1^ chloramphenicol overnight at 37°C. The following day, the colonies were used to inoculate 20 ml of fresh LB medium and the pre-cultures were grown overnight at 37°C shaking at 220 rpm. The next day, 200 ml of LB medium with antibiotics was inoculated with 20 ml of pre-cultures and bacteria were grown at 37°C shaking at 220 rpm. After reaching an OD_600_ of 0.6, protein expression was induced with 0.1 mM IPGT and bacteria were incubated in a shaker at 18°C for ~ 17h. Subsequently, the cells were harvested, pelleted and re-suspended in 2 ml lysis buffer (50 mM NaH_2_PO_4_, 300 mM NaCl and 10 mM imidazol, pH 8.0). Following lysozyme (Sigma Aldrich, Germany) (1 mg ml^-1^ final concentration) and protease inhibitor addition, samples were incubated for 30 min on ice. After additional cell disruption by 4 freeze-thaw cycles, RNAse A and DNAse were added to cell suspension following incubation on ice for 15 min. Next, 10 μl of Triton-X (Sigma-Aldrich, Germany), 600 μl of 5M NaCl and 100 μl of glycerol were added. The lysate was centrifuged and His-tagged proteins were purified with Ni-NTA resin (Qiagen, Hilden, Germany) according to manufacturer’s instructions. Wash buffer (pH 8.0) contained 50 mM NaH_2_PO_4_, 300 mM NaCl, and 50 mM imidazole and elution buffer (pH 10.2) contained 50 mM NaH_2_PO_4_, 300 mM NaCl, and 250 mM imidazole.

Purified protein concentrations were determined fluorometrically using Qubit 3.0 Fluorometer (ThermoFisher Scientific, USA). The recombinant proteins were analyzed on 5%–10% SDS-PAGE using Mini-PROTEAN II Electrophoresis Cell (BioRad, USA) followed by Coomassie blue staining and immuno-blot. His-probe antibody in 1:100 dilution (H-3. sc-8036, Santa Cruz Biotechnology, USA) and goat anti-mouse IgG-HPR (1:5,000, Agrisera Antibodies, Sweden) were used to confirm the presence of 6xHis labeled proteins. The bound antibodies were visualized by enhanced chemiluminescence (ECL). Radiographic film (Kodak X-Omat LS, Sigma-Aldrich, USA) exposure was performed for 10 min for detection.

### *In vitro* enzymatic assays

The activity of the recombinant PRISEs was tested based on the consumption of the putative substrate 8-oxogeranial and the formation of the reaction product *cis,trans*-nepetalactol. The enzyme assay was conducted following the protocol from Sherden et al. (2017), and it contained 50 mM MOPS (pH 7.5), 100 mM NaCl, 1 mM NADPH, tetrahydrofuran (THF) (0.5% v/v), 500 μM 8-oxogeranial (Santa Cruz Biotechnology, USA), and 2.5–5 mg of recombinant ISY. A negative control was conducted without the enzyme. The reactions were incubated overnight at 30°C, followed by extraction with 1:1 (v:v) hexane. Following enzymatic assay for the confirmation of *Nr*PRISE2 and *Nn*PRISE function, reaction products were subjected to GC/MS, UHPLC/(+)MS2, and NMR analyses, for the confirmation of their structure.

### GC/MS targeted analysis of 8-oxogeranial and cis,trans-nepetalactol

Pure standards of 8-oxogeranial (Santa Cruz Biotechnology, Dalas, Texas, USA) and *cis*,*trans*-nepetalactol (SigmaAldrich, St. Louis, MO, SAD) diluted in *n*-hexane (1 mg ml^-1^), reaction mixtures containing *Nr*PRISE2 and *NnPRISE*, and those lacking recombinant enzymes, were analyzed using GCMS-QP2010 plus instrument (Shimadzu, Japan) equipped with a split-splitless injector. Gas chromatographic separations was achieved adopting a ZB-1 MS column (30 mm × 0.25 mm, 0.25 μm film thickness) (Phenomenex, Austria), and using He (99.999%, The Linde Group, Ireland) as a carrier gas at a flow rate of 1.6 ml min^−1^. Transfer line and detector temperatures were maintained at 250°C and 270°C, respectively. Mass spectra were acquired in positive EI mode (+70 eV), with temperature of the EI source set to 280°C. The temperature program of the chromatographic oven was set to linearly increase the temperature from 50 to 240°C, at rate of 5°C min^−1^, and then maintain 240°C for the next 10 min. The splitless mode was applied for injection of samples (2 μl). Analyses were performed in SCAN mode, tracking the compounds within the range 40 to 400 amu, and in Single Ion Monitoring mode (SIM) which was targeted towards masses corresponding to 8-oxogeranial (166) and nepetalactol (168). The constituents of the reaction mixtures were identified by comparison of their mass spectra and retention times with those of the respective standards, and by comparison with the Wiley8, NIST05, and FFNSC3 libraries, using different search engines.

### NMR analysis of PRISE reaction products

Detection and structure confirmation of targeted compounds was performed using ^1^H and 2D H−H *COSY* NMR techniques and comparing the obtained spectral data with in house data of pure compounds. The NMR spectra were recorded on a Bruker AVANCE III 500 MHz NMR spectrometer equipped with a 5 mm inverse broadband (BBI) probe head at 298 K. Samples were dissolved in 500 µL of 99.8% CDCl_3_ (SigmaAldrich, Germany) with 0.03% (v/v) of internal standard trimethylsilane (TMS).

To generate ^1^H NMR spectra, 32k data points were collected using standard pulse program *zg30* with 128 scans. Spectral width was set to 20 ppm (10,005.2 Hz), relaxation time to 2 sec (d1), acquisition time 2 sec, and transmitter frequency offset to 8.5 ppm. Spectral referencing was performed used chemical shift of TMS, set to *δ* = 0 ppm). Total acquisition time was 10 min. For the 2D H−H *COSY* NMR spectra, spectral width was set to 5,502.8 HZ for F1 and F2 frequency axis, relaxation time 2 sec and frequency offset to 5.75 ppm. Spectra were acquired using 32 scans per 256 increments of F1, with FID of 2k data points per F2. Total duration of analyses was 90 min.

### Evolutionary analysis of *Nr*PRISEs and *Nn*PRISE by maximum likelihood method

The evolutionary history was inferred by using the Maximum Likelihood method and JTT matrix-based model (Jones et al., 1992). Phylogeny test was conducted by bootstrap method and number of bootstrap replications were 1,000. The tree with the highest log likelihood is shown. The percentage of trees in which the associated taxa clustered together is shown next to the branches. Initial tree(s) for the heuristic search were obtained automatically by applying Neighbor-Join and BioNJ algorithms to a matrix of pairwise distances estimated using the JTT model, and then selecting the topology with superior log likelihood value. The tree is drawn to scale, with branch lengths measured in the number of substitutions per site. This analysis involved 38 amino acid sequences. All positions containing gaps and missing data were eliminated (complete deletion option). There were a total of 344 positions in the final dataset. Evolutionary analyses were conducted in MEGA X (Kumar et al., 2018).

### *Nr*PRISE2 and *Nn*PRISE tertiary structure modeling and ligand docking

The tertiary structure of ISY and PRISE proteins was predicted with AlphaFold2.1 (Jumper et al., 2021) via UCSF ChimeraX 1.4 (Pettersen et al., 2021). Assessment of obtained structures was performed via SWISS-MODEL Workspace (Waterhouse et al., 2018) by MolProbity 4.4 (Williams et al., 2018). The obtained PDB structures ware compared to ISY/P5βR enzymes with experimentally resolved 3D structures using the jFATCAT rigid model (Li et al., 2020) and in ChimeraX after superposition using the matchmaker command with default parameters (Pettersen et al., 2021).

Cofactor (NADP) inclusion in the predicted ISY structures was performed by first superposing of *Digitalis lanata* P5βR (PDB:2V6G, Thorn et al., 2008) using the matchmaker command in ChimeraX after which the cofactor atom coordinates were extracted and the predicted ISY protein-cofactor complex energy was minimized using Gromacs 2018.6 (Abraham et al., 2015). For energy minimization the protein topology was prepared using the CHARMM36 all-atom force field (Huang and Mackerell, 2013, jul 2021 version, http://mackerell.umaryland.edu/charmm_ff.shtml#gromacs); ligand topology was generated using CHARMM General Force Field server (Vanommeslaeghe et al., 2009, https://cgenff.umaryland.edu/initguess/). Solvation of the complex was performed using the CHARMM-modified TIP3P water model (*TIP3P*_CHARMM), while energy minimization of the complex was performed using steepest descent algorithm with 50k minimization steps using the following options: emtol = 50.0, emstep = 0.01, nstlist = 1, cutoff-scheme = Verlet, ns_type = grid, rlist = 1.2, coulombtype = PME, rcoulomb = 1.2, vdwtype = cutoff, vdw-modifier = force-switch, rvdw = 1.2, pbc = xyz, DispCorr = no.

The cofactor-protein energy minimized structures were used for ligand docking using AutoDock Vina 1.2.3 (Eberhardt et al., 2021). For docking the following ligands were used: 8-oxogeranial, *trans*- and *cis*- 8-oxocitronellyl enolates, (1R)-*c*,*c*-NLL (PubChem CID: 11194562), (1R)-*c*,*t*-NLL (PubChem CID: 442438), (1S)-*c*,*c*-NLL (PubChem CID: 11298185), (1S)-*c*,*t*-NLL (PubChem CID: 11286692), (1S)-*t*,*c*-NLL and (1R)-*t*,*c*-NL (obtained by reducing *t*,*c*-NL (PubChem CID: 442430) using Avogadro 1.2.0 (Hanwell et al., 2012). 50 conformers of each of the bicyclic ligands was generated via RDKit 2021.09.5 (Landrum et al., 2022) using the ETKDG version 3 method with small ring torsion angle preferences (Wang et al., 2020) and subsequently filtered using an RMSD threshold of 0.5 Å so that only those conformations that are at least 0.5 Å RMSD away from all retained are kept. This resulted in 2–4 conformers per compound. The geometry of the resulting conformers was optimized using Merck molecular force field (MMFF94s) as implemented in RDKit 2021.09.5 (Landrum et al., 2022). 8-Oxogeranial ligand was prepared so that Cα-Cβ bonds were held rigid in the following conformations: 1. C1-C2 s-*cis*, C7-C8 s-*cis*, 2. C1-C2 s-*cis*, C7-C8 s-*trans*, 3. C1-C2 s-*trains*, C7-C8 s-*cis* and 4. C1-C2 s-*trains*, C7-C8 s-*trans*. Two types of ligand dockings were performed: using rigid protein structures and flexible docking where the residues Lys147 and Tyr179 for *Nn*PRISE and Phe153 and Tyr185 for *Nr*ISY were allowed to be flexible. The docking procedures were performed using an exhaustiveness of 64, with autogrid4 precalculated affinity maps (Hanwell et al., 2012). 8-Oxogeranial coordinates from the *C. roseus* ISY (Qin et al., 2016, PDB: 5COB) were used to define the docking box center, after superposition to the AlphaFold models of *Nn*PRISE (grid center: x = 7.484, y = -4.133, z = 3.346) *and Nr*ISY (grid center: x = 6.686, y = -7.040, z = 2.655). The docking box was 40 grid points in each direction, with a grid spacing of 0.375 Å. The highest scoring poses for each ligand were inspected and compared to 8-oxogeranial from the experimental structures of *C. roseus* ISY – PDB: 5COB (Qin et al., 2016) and PDB: 5DBI (Hu et al., 2015).

8-Oxogeranial α,β -conformer energy evaluation and minimization was performed with Avogadro 1.2 (Hanwell et al., 2012) using steepest descent algorithm and the following forcefields: Universal force field (UFF, Rappe et al., 1992), Merck molecular force fields - MMFF94 (Halgren, 1996) and MMFF94s (Halgren, 1999) and the general Amber force field (GAFF, Wang et al., 2004).

### Statistical analysis

For the Hierarchical Cluster Analysis (HCA) the input variables were scaled to the [0, 1] range. HCA was based on Pearson method of cluster agglomeration, adopting the Morpheus software (https://software.broadinstitute.org/morpheus). The correlation matrix for the gene expression quantitative data was constructed using Pearson’s correlation coefficients, with the Past 4 software (version 4.12; Hammer et al., 2001). Quantitative metabolomics and gene-expression data were subjected to *post hoc* Tukey’s test (p < 0.05) of one way ANOVA, or to Student’s *t-*tests (p < 0.05).

## Results and discussion

### Untargeted metabolomics of *N. rtanjensis* and *N. nervosa* leaves

The present study describes for the first time the comprehensive profiling of iridoids in *N. rtanj*ensis and *N. nervosa*, by simultaneously acquiring and comparatively analyzing iridoid aglycones and glycosides. Methanol extracts of *N. rtanjensis* and *N. nervosa* leaves, harvested from *in vitro* grown plants, were subjected to non-targeted metabolomics adopting GC/MS for the analysis of iridoid aglycones, and UHPLC/QToF MS^2^, in both negative and positive ionization modes, for the analysis of iridoid glycosides and iridoid aglycones. Currently available data on the distribution and diversity of these two subgroups of iridoids across the genus *Nepeta* are fragmentary; only rarely they are simultaneously analyzed. Complementary analytical methodologies and tools, described within the present study, could easily be adopted to other *Nepeta* species, which can facilitate the elucidation of the overall diversity of iridoids at inter- and intra-species level, and further direct the reconstruction of the molecular background of this diversity within the genus.

GC/MS analysis of methanol extracts revealed the presence of a variety of terpenoids in *N. rtanjensis* and *N. nervosa* leaves (**Supplementary Table 2**). The total number of identified compounds in *N. rtanjensis* and *N. nervosa* leaves was 26 and 10, respectively. In leaves of *N. rtanjensis*, the most abundant were monoterpenoids from the group of iridoid aglycones, 5,9-dehydronepetalactone and *trans*,*cis*-nepetalactone, which were followed by *cis,trans*-nepetalactone (**Supplementary Table 2**; **Figure 1**). Germacrene D was the major sesquiterpenoid in methanol extracts of *N. rtanjensis*, while monoterpenoids *α*-thujene and *α*-copaene were also present in significant amounts. Although iridoids were not identified in methanol extracts of *N. nervosa* leaves, this species contained significant amounts of sesquiterpenoids, among which Germacrene D and Germacrene D-4-ol predominated (**Supplementary Table 2**). Diterpene phytol was also abundant in leaves of *N. nervosa*.

UHPLC/(+)QToF MS^2^ analysis in the positive ionization mode confirmed the presence of the two nepetalactone diasteroisomers and 5,9-dehydronepetalactone in methanol extracts of *N. rtanjensis* leaves, and identified several more iridoid aglycones (**Supplementary Table 3**). Dihydronepetalactone belongs to the group of iridoid aglycones and is a hydrogenation product of nepetalactone, while nepetaracemoside B aglycone and 7-deoxyloganin aglycone most likely arise through the deglycosilation of nepetaracemoside B and deoxyloganin, two compounds which are a part of the biosynthetic branch leading to iridoid glycosides. Two stereoisomers of nepetalactone, *cis*,*trans*- and *trans*,*cis*-nepetalactone, and 5,9-dehydronepetalactone, were very abundant in *N. rtanjensis* leaves, which is in accordance with previously published data (Mišić et al., 2015; Skorić et al., 2017; Aničić et al., 2021). By studying the exact mass and high-resolution MS^2^ fragmentation of iridoid glycosides in the negative ionization mode, 6 compounds (16-21) specific to the *Nepeta* genus were identified in *N. rtanjensis* leaves, as well as 5-deoxylamiol (15), previously found in other Lamiaceae species. A total of 13 iridoid glycosides have previously been found in *N. rtanjensis* cultured *in vitro* (Aničić et al., 2021), including 1,5,9-*epi*deoxyloganic acid, geniposide, 1-*O*-hexosyl-epideoxyloganic acid, and nepetariaside, also identified within the present study (**Supplementary Table 2**). 1,5,9-*epi*deoxyloganic acid is recognized as the major iridoid glycoside in *N. rtanjensis*, and was previously identified as one of the major compound from this subgroup in other *Nepeta* species, including *N. cataria* (Murai et al., 1984), *N. cadmea* (Takeda et al., 1998), *N. nuda* (Kökdil et al., 1998; Petrova et al., 2022), and *N. argolica* (Ahmed et al., 2006). Interestingly, no iridoids were identified in methanol extracts of leaves of *in vitro* grown *N. nervosa* plants (**Supplemetary Tables 1** and **2**; **Figure 1**). Literature search revealed only a few publications dealing with the chemical profiling of *N. nervosa* (Nestorović et al., 2010; Mišić et al., 2015), which pointed to the fact that this species produces no iridoids. However, it should not be ruled out that this species actually produces some of the iridoids in amounts which are below the limits of detection of the adopted analytical instruments. It could also be speculated that *N. nervosa* plants might produce iridoids under more favorable growth conditions.

In addition to iridoids, compounds belonging to the class of phenolics (14 compounds) and other classes (10 compounds) were also identified in analyzed samples (**Supplementary Table 3**). Hydroxycinnamic acids (10 compounds) were represented by glycosides and esters of caffeic and ferulic acids. Rosmarinic acid, which is structurally a dimer of caffeic acid, was abundant in leaves of both analyzed *Nepeta* species, while 5-*O*-caffeoylquinic acid and 3-O-caffeoylquinic acid were present only in *N. rtanjensis*. Nepetoidin A or B, a derivative of caffeic acid specific for plants from the genus *Nepeta* (Aničić et al., 2021), was identified in leaves of *N. nervosa*. Totally 4 compounds from the group of flavonoids are identified in leaves of *N. nervosa*. Two aglycones (13 and 14), which are methylated flavones according to their structure, are specific for the genus *Nepeta* (Aničić et al., 2021). Luteolin 7-*O*-dihexuronide (11), detected at 5.59 min, showed molecular ion at 637.10464 *m/z* and MS^2^ base peak at 285.03234 *m/z* (deprotonated luteolin). MS^2^ fragments at 175 and 193 *m/z* originate from hexuronic acid, while the ion found at 351 *m/z* corresponds to the fragment resulting from the neutral loss of the luteolin molecule, which is the proof that 2 molecules of hexuronic acid are interconnected (Dienaitė et al., 2018). For compound 12 at 8.38 min and 577.15518 *m/z*, the proposed molecular formula was C_27_H_29_O_14_^+^, based on the correct isotopic mass. The MS^2^ base peak at 329.10012 *m/z* was formed by the neutral loss of C_9_H_12_O_8_ (248 Da), which by mass completely corresponds to the malonyl-hexose residue. Such a compound has not been found in the genus *Nepeta* so far, nor in any other plant species. However, a similar compound without malonyl group was detected in *Salvia* species (Qiao et al., 2009), and it is known as salvigenin 5-*O*-glucoside.

### Comparative transcriptomics of *N. rtanjensis* and *N. nervosa* leaves

Transcriptomes of *N. rtanjensis* and *N. nervosa* leaves were searched for the presence/absence of transcripts of iridoid pathway-related genes, based on the homology with the genes characterised in iridoids- and alkaloids- producing *C. roseus* (Geu-Flores et al., 2012; Miettinen et al., 2014; Krithika et al., 2015) and those from the genus *Nepeta* (Lichman et al., 2019a; Lichman et al., 2020; Hernández Lozada et al., 2022). This resulted in the list of candidate biosynthetic genes from *N. rtanjensis* and *N. nervosa*. In the transcriptome of *N. rtanjensis* leaves, orthologues of *NrGPPS*, *NrGES*, *NrG8H*, *Nr8HGO*, *NrPRISE1*, *NrPRISE2*, *NrNEPS1*, *NrNEPS2*, *NrNEPS3, NrNEPS4*, and *NrMLPL1* and *NrMLPL2* were identified (Aničić et al., 2020) (**Figures 1B, C**). Due to their affiliation with the “promiscuous” enzyme group – PRISE (Petersen et al., 2016), the potential ISY orthologs were labeled as the PRISE enzymes for the purpose of initial functional characterization and co-expression analyses. The designations include *Nr*PRISE1, *Nr*PRISE2, and *Nn*PRISE. As *N. rtanjensis* is predominately a *trans*,*cis*-nepetalactone producing species, it is not surprising that NEPS4 and NEPS1 homologues were identified in the transcriptome of leaves of this species. It was previously shown that NEPS4 enzymes in *N. cataria* and *N. mussinii* are *trans*-*cis*-cyclazes, which act with a partner dehydrogenase NEPS1 and ISY to give a rise to *trans*,*cis*-nepetalactone (Lichman et al., 2020; Hernández Lozada et al., 2022). On the other hand, NEPS1 has also been reported to have *cis,trans*-nepetalactol dehydrogenase activity (Lichman et al., 2019a). *N. rtanjensis* leaves also contain significant amounts of *cis,trans*-nepetalactone, and their transcriptome is shown to possess homologue of NEPS2, which in *N. sibirica* (*Ns*NEPS2) acts as a *cis*-*trans* cyclase and oxidase (Lichman et al., 2019a; Hernández Lozada et al., 2022). According to the literature data, NEPS3 has specific 7*S*-*cis,cis-*nepetalactone cyclase activity (Lichman et al., 2019a).

Orthologues of *GPPS*, *G8H*, *8HGO*, *PRISE*, *MLPL*, and *NEPS* were present in the transcriptome of *N. nervosa* leaves, while GES transcripts were lacking. Interestingly, only one orthologue of both PRISE (*NnPRISE*) and NEPS (*NnNEPS1*) were identified in *N. nervosa*. The reaction catalyzed by GES is considered a key gatekeeping step in iridoid biosynthesis, since this enzyme is responsible for diverting metabolic flux away from canonical monoterpenes by converting GPP to geraniol, the precursor of iridoids (Boachon et al., 2018). Silencing of GES in *N. cataria* leaves resulted in significant decrease of the content of all nepetalactoneL isomers and 1,5,9-*epi*deoxyloganic acid (Palmer et al., 2022), while in *C. roseus* the content of iridoid-derived alkaloids was decreased (Kumar et al., 2015). As NEPS and MLPL work in combination with ISY to control the stereochemical mode of 8-oxogeranial cyclization (Lichman et al., 2020), it might be speculated that besides GES, functional ISY and some of the NEPSs are important missing parts to complete the iridoid biosynthesis puzzle in this species. The loss of function of NEPSs, which are directly responsible for nepetalactone formation and stereochemistry at the 4a and 7a carbons (Lichman et al., 2019a; Lichman et al., 2019b), can certainly lead to the absence of nepetalactones in these *Nepeta* species.

We further compared the protein sequences corresponding to iridoid-related biosynthetic genes of the two *Nepeta* species. Pairwise sequence alignment results revealed high level of similarity between the homologues of all identified iridoid-related biosynthetic genes of the two species (**Figure 1C**). MLPL sequences had shown lowest similarity between the two species, while *Nn*PRISE displayed especially high sequence similarity with *Nr*PRISE1 (96.4%).

### Phyllogenetic analysis of *Nr*PRISE2 and *Nn*PRISE

**Figure 2:**
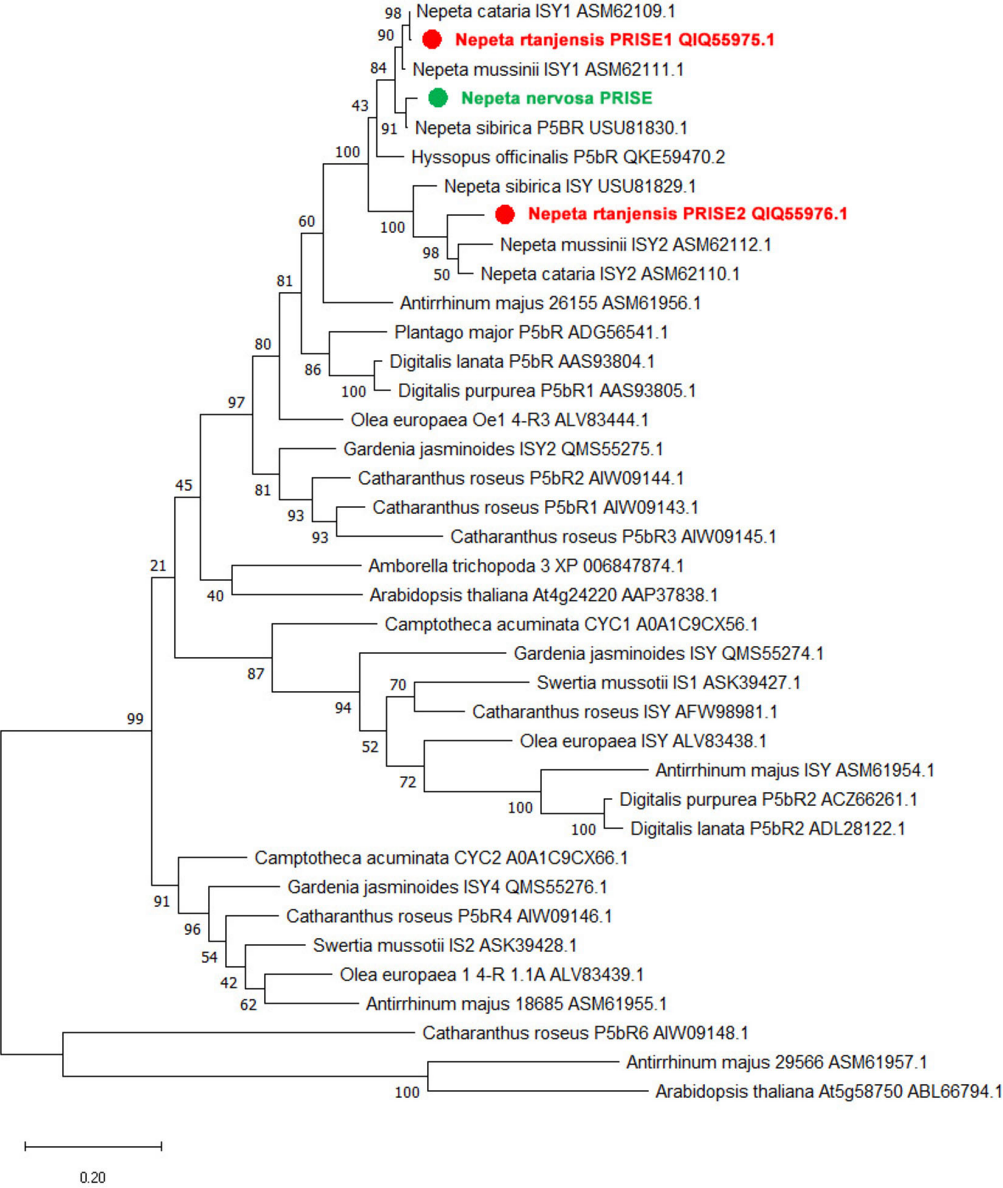
The Maximum Likelihood tree generated with MEGA X (version 10.2.6) illustrating the phylogenetic relationship of NrPRISE1, NrPRISE2, and NnPRISE candidates compared to homologous genes from different plant species. The tree with the highest log likelihood is shown. The percentage of trees in which the associated taxa clustered together is indicated next to the branches. Accession numbers of sequences are listed next to species names and protein abbreviations.

The nucleotide sequences potentially encoding ISYs found in leaf transcriptomes of *N. rtanjensis* – *NrPRISE1* (1164 bp) and *NrPRISE2* (1203 bp), and of *N. nervosa* - *NnPRISE* (1167 bp), have shown to be highly similar to functionally characterized ISYs from other species (**Figure 2**). The *Nr*PRISE2 is most closely related to the characterized iridoid synthases from *N. cataria* (*Nc*ISY) and *N. mussinii* (*Nm*ISY) (Lichman et al., 2020). Recombinant *Nc*ISY and *Nm*ISY enzymes were shown to be active isoforms, which fully converted 8-oxogeranial to *cis,trans*-nepetalactol in assays *in vitro* (Sherden et al., 2017; Lichman et al., 2020). On the other hand, *Nn*PRISE and *Nr*PRISE1 are more similar to P5βRs isoforms from *N. cataria* (*Nc*P5βR1) and *N. mussinii* (*Nm*P5βR1). P5βR isoforms display very low catalytic activity (Sherden et al., 2017), which is why they are thought not to be physiologically relevant for iridoid biosynthesis (Lichman et al., 2020).

Although phylogenetic relationships of the previously characterized ISYs and PRISE candidates from *N. rtanjensis* and *N. nervosa* indicate a high degree of mutual homology, this information alone cannot predict with absolute certainty the catalytic activity of the enzymes. Furthermore, ISYs belong to the “promiscuous” group of enzymes, together with progesterone-5-β-reductases (PRISE) (Petersen et al., 2016), that are structurally and functionally very close, which additionally makes unreliable the prediction of isolated isoforms activity solely based on the phylogenetic analysis. Therefore, we proceed with the organ-specific and MeJA-induced profiling of iridoids content and expression profiling of corresponding biosynthetic genes to further support the phylogenetic data.

### Organ-specific and MeJA-induced patterns of iridoid biosynthesis in *N. rtanjensis* and *N. nervosa*

**Figure 3:**
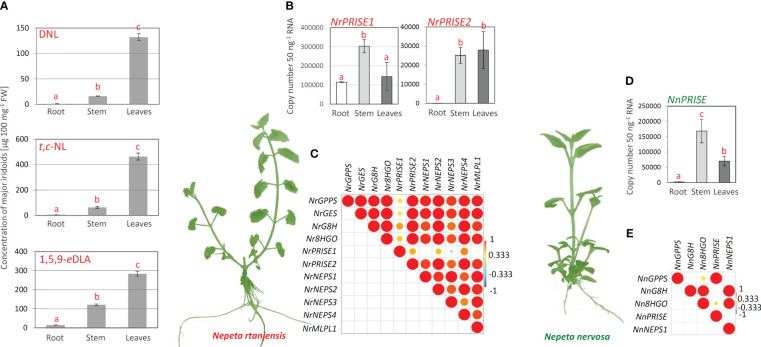
**(A)** Organ-specific profiling of the major iridoid aglycones and iridoid glycosides in *N. rtanjensis* plants grown *in vitro* was performed adopting UHPLC/DAD/(±)HESI−MS^2^ analysis. No iridoids were recorded in analyzed *N. nervosa* samples. Error bars represent SE of three biological replicates. In all cases, bars with different letters are significantly different (p < 0.05) according to *post hoc* Tukey’s test of one way ANOVA. **(B)** Expression profiles of *N. rtanjensis* PRISE candidates (*NrPRISE1* and *NrPRISE2*) and **(D)** *N. nervosa NnPRISE* in roots, stems and leaves of *in vitro* grown plants. Letters above the bars denote significant differences according to Tukey’s HSD *post hoc* test at p < 0.05. Pearson correlations based on the relative gene expression of iridoid-related biosynthetic genes in **(C)** *N. rtanjensis* and **(E)** *N. nervosa*. Each field in the Pearson’s correlation plot indicates the coefficient for a pair of genes, and the value of the correlation coefficient is represented by the intensity of red and yellow (positive correlations) or blue and green (negative correlations), as indicated on the color scales (for the interpretation of the references to color in this Figure legend, the reader is referred to the web version of this article). GPPS, geranyl diphosphate synthase; GES, geraniol synthase; G8H, geraniol 8-hydroxylase; 8HGO, 8-hydroxygeraniol oxidoreductase; PRISE, progesterone-5β-reductase/iridoid synthase activity displaying enzymes; NEPS, nepetalactol-related short-chain dehydrogenase; MLPL, major latex protein-like enzyme; IA, iridoid aglycone; IG, iridoid glycoside; DNL, 5,9-dehydronepetalactone; *t,c*-NL, *trans,cis*-nepetalactone; 1,5,9-*e*DLA, 1,5,9-*epi*deoxyloganic acid.

According to the organ-specific profiling of major iridoids in *N. rtanjensis* shoots grown *in vitro*, leaves displayed the highest content of *trans*,*cis*-nepetalactone, 5,9-dehydronepetalactone, and 1,5,9-*epi*deoxyloganic acid, and were followed by stems (**Figure 3A**). Roots contained no targeted iridoids. It has previously been shown that leaves of *N. rtanjensis* are enriched with glandular trichomes, the main site of nepetalactone biosynthesis and accumulation in *Nepeta* species (Aničić et al., 2018; Aničić et al., 2020). In parallel with iridoid profiling, we analyzed co-expression patterns of iridoid biosynthesis-related gene candidates (*NrGPPS*, *NrGES*, *NrG8H*, *Nr8HGO*, *NrPRISE1*, *NrPRISE2*, *NrNEPS1*, *NrNEPS2*, *NrNEPS3, NrNEPS4*, and *NrMLPL*) in the same organs. Leaves and stems were more enriched with the transcripts of the majority of analyzed biosynthetic genes candidates when compared to roots. The expression of *NrPRISE2* in roots was very low, while in *N. rtanjensis* leaves around 400 fold higher amount of *NrPRISE2* transcripts was recorded (**Figure 3B**). As for *NrPRISE1*, the highest expression level of this PRISE orthologue was recorded in stems of *N. rtanjensis* (**Figure 3B**). Expression profiles of selected iridoid-related biosynthetic gene candidates in leaves, stems and roots of *N. rtanjensis* were subjected to the Pearson’s correlation analysis. Results indicate that the majority of the analyzed genes are co-expressed while displaying significant positive correlations (**Figure 3C**). The only exception was *NrPRISE1*, which displayed the lowest level of correlations with other gene candidates. The majority of genes analyzed within the present study (*NrGPPS*, *NrGES*, *NrG8H*, *Nr8HGO*, *NrPRISE1*, *NrPRISE2*, *NrNEPS1*, *NrNEPS2*, *NrNEPS3*, and *NrNEPS4*) were previously analyzed for their expression profiles in detached trichomes and abraded leaves (Aničić et al., 2020), but also in response to PEG-induced dehydration of leaves (Aničić et al., 2020). All these data point to the *NrPRISE2* as a promising candidate for iridoid synthase, an enzyme involved in the reduction of nepetalactol, a critical point determining metabolic flux throughout the iridoid pathway. *N. rtanjensis NrPRISE2* orthologue was further subjected to functional characterization.

Nepetalactone, 5,9-dehydronepetalactone, and 1,5,9-*epi*deoxyloganic acid were not recorded in leaves, stems, and roots of *N. nervosa* grown *in vitro*, as expected (Nestorović et al., 2010; Mišić et al., 2015). No literature data on the organ- or tissue-specific iridoid profiling is available for *N. nervosa.* Organ-specific expression patterns of selected biosynthetic gene candidates (*NnGPPS*, *NnG8H*, *Nn8HGO*, *NnPRISE*, *NrNEPS1*, and *NnMLPL*) were also analyzed in *N. nervosa*. The lowest expression level of the analyzed genes was recorded in roots of *N*. *nervosa*, while stems and leaves were enriched with transcripts of targeted biosynthetic gene candidates. Of the analyzed genes, *NnPRISE* displayed ~2 fold higher expression in leaves than in roots (**Figure 3D**), as well as the lowest level of positive correlations with other analyzed genes (**Figure 3E**). Pearson’s correlation analysis showed that the expression of *NnPRISE* is significantly positively correlated with the *NnGPPS*, while no significant correlations were observed with other analyzed genes (**Figure 3E**). Some of the BG candidates are co-expressed in different tissues (*Nn*GPPS with *Nn*PRISE, as well as *Nn*NEPS1 with *Nn*G8H and *Nn*8HGO), indicating their possible involvement in the iridoid biosynthetic pathway.

**Figure 4:**
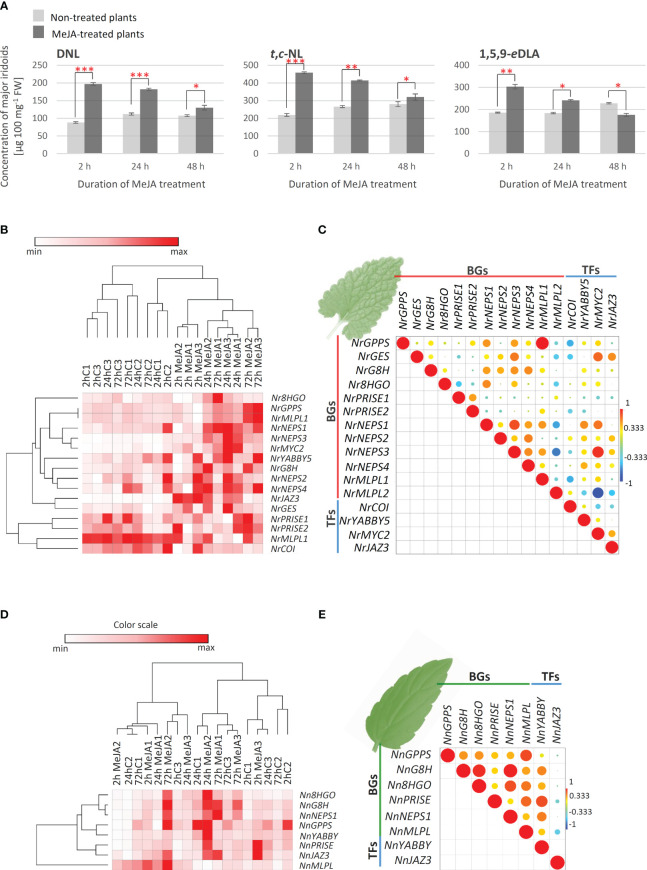
**(A)** MeJA- induced changes in the content of major iridoids in *N. rtanjensis* leaves as quantified using UHPLC/DAD/(±)HESI−MS2 instrument: DNL − 5,9-dehydronepetalactone; *t*,*c*-NL − *trans*,*cis*-nepetalactone; 1,5,9-*e*DLA − 1,5,9-*epi*deoxyloganic acid. Error bars represent SE of three biological replicates. In all cases, bars with different letters are significantly different (P < 0.05) according to *post hoc* Tukey’s test of one way ANOVA. Red asterisks denote significantly different values according to the *t*-test, *p*-values, *p< 0.05, **p< 0.01, ***p< 0.001. Heatmap of the scaled quantitative data (expression data of iridoid-related biosynthetic genes- BGs and transcription factors- TFs) in *N. rtanjensis* **(B)** and *N. nervosa* leaves (**D**), with the samples (both columns and rows) arranged according to the hierarchical cluster analysis (Pearson method of cluster agglomeration). Intensity of red color indicates the expression levels of targeted genes, with red color representing the max values and white color the min values recorded for individual gene, as indicated in the color scale. Pearson correlation plot based on the gene expression of biosynthetic genes and transcription factors in leaves of **(C)** *N. rtanjensis* and **(E)** *N. nervosa* is presented. Each field in the Pearson’s correlation plot indicates the coefficient for a pair of genes, and the value of the correlation coefficient is represented by the intensity of red and yellow (positive correlations) or blue and green (negative correlations), as indicated on the color scales (for the interpretation of the references to color in this Figure legend, the reader is referred to the web version of this article). GPPS, geranyl diphosphate synthase; GES, geraniol synthase; G8H,geraniol 8-hydroxylase; 8HGO, 8-hydroxygeraniol oxidoreductase; PRISE, progesterone-5β-reductase/iridoid synthase activity displaying enzymes; NEPS, nepetalactol-related short-chain dehydrogenase; MLPL, major latex protein-like enzyme; COI, coronatine-insensitive transcription factor; JAZ3, jasmonate ZIM-domain 3; MYC2, transcription factor Mouse-ear cress 2; YABBY5, YABBY5 transcription factor; DNL, 5,9-dehydronepetalactone; *t,c*-NL, *trans,cis*-nepetalactone; 1,5,9-*e*DLA, 1,5,9-epideoxyloganic acid.

To elucidate the interrelation between *N. rtanjensis* and *N. nervosa* metabolome and transcriptome, the effect of methyl jasmonate (MeJA) as a potent elicitor, on iridoid content and relative changes in the expression of biosynthetic gene candidates in leaves of *N. rtanjensis* and *N. nervosa* grown *in vitro*, was investigated. MeJA has previously been used as a potent elicitor of specialized metabolism in plants, including those producing iridoids (e.g. (Wang et al., 2010; Cao et al., 2016; Piątczak et al., 2016; Matekalo et al., 2018; Rubio-Rodríguez et al., 2021; Pu et al., 2022). This elicitor promotes changes in the accumulation of specialized metabolites at the transcriptional level of genes involved in their biosynthesis. Content of major iridoids was significantly increased in MeJA-treated *N. rtanjensis* plants 24 h following the application of the elicitor (**Figure 4A**). The most significant increase was recorded for 5,9-dehydronepetalactone, which was followed by *trans*,*cis*-nepetalactone and 1,5,9-*epi*deoxyloganic acid. Significantly higher amounts of 5,9-dehydronepetalactone and *trans*,*cis*-nepetalactone, and lower amounts of 1,5,9-*epi*deoxyloganic acid were also recorded in MeJA-treated plants than in non-treated plants 72 h after the beginning of the experiment (**Figure 4A**). The highest increase in the expression of the majority of targeted biosynthetic genes and transcription factors candidates was recorded 24 h after the application of MeJA, and after that the expression decreases (**Figure 4B**). Still, the expression of the majority of biosynthetic genes and transcription factors is higher in MeJA-treated than in non-treated plants after 72 h of experiment. The changes in the expression levels were the most pronounced for *NrNEPS3* and transcription factors *NrJAZ3* and *NrMYC2*. Similarly, in *Castilleja tenuifora* the increase in the expression of some iridoid biosynthesis-related genes was the most pronounced 24 h after the MeJA treatment (Rubio-Rodríguez et al., 2021). The selection of TF for the co-expression analysis was based on our previous studies, where NrMYC2 and NrYABBY5 are recognized as the potential positive regulators of NL biosynthesis in *N. rtanjensis* (Aničić et al., 2020). *NrPRISE1 and NrPRISE2* clustered close to *NrMLPL2* and *NrCOI1* on Pearson HCA matrix (**Figure 4B**). All the other analyzed genes formed a separate cluster, which indicates their coordinated changes in the expression levels. To study the interrelation between the expression profiles of the iridoid-related biosynthetic genes and transcription factors in MeJA-treated leaves, a Pearson’s correlation analysis was performed (**Figure 4C**). The expression levels of the majority of analyzed genes were positively correlated, with the exception of *NrMLPL2, NrCOI1*, *and NrPRISE1* which displayed mainly negative correlations with the rest of analyzed genes (**Figure 4C**). Although statistically significant positive correlations were observed in several cases (**Figure 4C**), the results show no clear co-expression patterns of analyzed *N. rtanjensis* genes, but point to the involvement of *Nr*JAZ and *Nr*MYC2 in jasmonate signaling pathway and indicate direct transcriptional regulation of *Nr*GES and *Nr*NEPS1-*Nr*NEPS4 via the above mentioned transcription factors. Thus, our results indicate the involvement of some of the analyzed genes in the same biosynthetic route, and point to the transcriptional regulation of their activity, which is consistent with our previous findings (Aničić et al, 2020). Our research suggested that shoot culture of *N. rtanjensis* in combination with MeJA elicitation has a promising application in biotechnology-assisted iridoid production.

As for iridoid non-producing *N. nervosa*, MeJA treatment did not induce the accumulation of iridoids in leaves. On the other hand, the expression of the majority of analyzed biosynthetic genes was induced 24h and 72h following the application of MeJA (**Figure 4D**). The most prominent changes were observed for *NnG8H*, *Nn8HGO* and *NnNEPS1*. One of the analyzed transcription factors, *NnYABBY*, clustered close to *NnGPPS* and *NnPRISE* on the Pearson HCA matrix (**Figure 4D**). *NnJAZ3* was segregated to the other analyzed biosynthetic genes. Pearson correlation analysis revealed positive correlations in the expression of all the analyzed biosynthetic genes and transcription factors, additionally indicating the transcriptional regulation of their activity (**Figure 4E**).

Based on the organ-specific and MeJA-induced profiling of expression of the PRISE candidates (*NrPRISE1*, *NrPRISE2*, and *NnPRISE*), their co-expression patterns with the rest of IBGs, as well as on the phylogenetic analysis, we further proceeded with functional characterization. As *Nr*PRISE1 showed no activity in enzymatic assays, we presumed that this PRISE candidate was not physiologically relevant for iridoid biosynthesis in *N. rtanjensis*, and we are further presenting the results of *NrPRISE2* and *NnPRISE* functional characterization.

### Functional characterization of recombinant NrPRISE2 and NnPRISE expressed in E. coli

**Figure 5:**
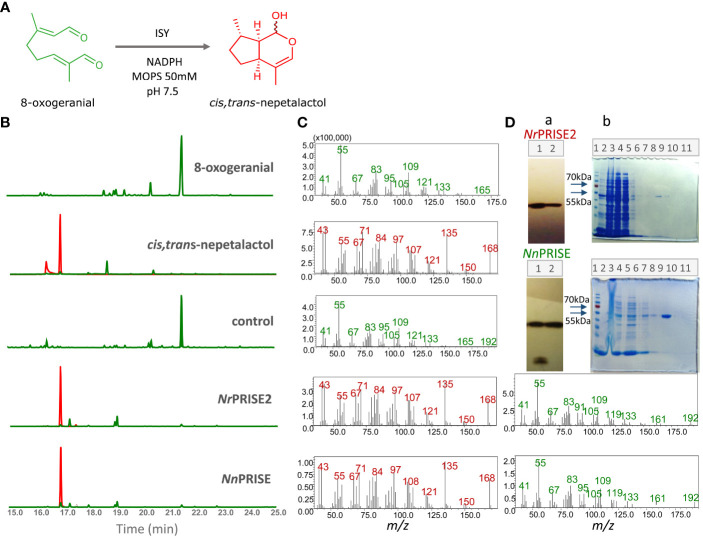
GC-MS analysis of *in vitro* assays with recombinant enzymes NrPRISE2 and NnPRISE. An enzymatic reaction is shown, in which iridoid synthase converts 8-oxogeranial to *cis,trans*-nepetalactol in a buffer with 50mM MOPS and pH 7.5 **(A)**. Extracted GC-MS chromatograms for 166 and 168 *m/z*, 8-oxogeranial and *cis,trans*-nepetalactol standards were compared with chromatograms of *in vitro* assays without enzymes (control) and with recombinant enzymes *Nr*PRISE2 and *Nn*PRISE **(B)**. The retention time of the peak corresponding to nepetalactol is Rt= 16.9 min, and 8-oxogeranial is Rt= 21.5 min. The peaks corresponding to the masses of 166 *m/z* are marked green, while the peaks of mass 168 *m/z* are red. The figure also shows the mass spectra **(C)** corresponding to the peaks from the chromatogram on the left with Rt= 16.9 min (red) and 21.5 min (green). Immunoblot **(A)** shows the isolated proteins *Nr*PRISE2 and *Nn*PRISE. 1 – insoluble proteins, 2 – soluble proteins **(D)**. The immunoblot figures have been extracted by cropping from the original images, constituting a part of **Supplementary Figure 5**. SDS-PAGE **(B)** shows the NI-NTA agarose protein purification process. 1- protein marker; 2 − proteins before IPGT introduction; 3 − insoluble proteins; 4 − soluble proteins; 5 − soluble proteins unbound to the NI-NTA resin; 6,7,8 − wash step 1, 2,3; 9, 10, 11 − elution steps 1,2,3. **(D)**. 8OG, 8-oxogeranial; NLL, *cis*,*trans*-nepetalactol; PRISE, progesterone-5β-reductase/iridoid synthase activity displaying enzymes.

SDS-PAGE analyzes and Coomassie Brilliant Blue staining, as well as immunoblot detection of recombinant *Nr*PRISE2 and *Nn*PRISE proteins with anti-His antibodies, confirmed the presence of a single major band of the expected monomer length of 42 kD (Geu-Flores et al., 2012; Sherden et al., 2017), in both *Nr*PRISE2 and *Nn*PRISE lanes (**Figure 5**). Recombinant *Nr*PRISE2 and *Nn*PRISE were biochemically characterized, adopting *in vitro* enzymatic assay to determine their catalytic activities (**Figure 5A**). Reaction mixtures with recombinant proteins, 8-oxogeranial as substrate, and a cofactor NADPH, as well as a control assay mixture that did not contain the enzymes, were extracted in hexane. Based on their accurate mass, elemental composition, and fragmentation pattern, the products were characterized as nepetalactol and iridodials using GC/MS and UHPLC/MS^2^ analyses (**Figures 5B, C**). *Cis,trans*-nepetalactol was visible as a peak at Rt = 16.9 min, while 8-oxogeranial was represented by a peak at Rt = 21.5 min on the GC/MS chromatogram (**Figure 5B**).

By comparing the GC/MS spectra and retention times of compounds from the reaction mixtures with those of pure 8-oxogeranial and *cis,trans*-nepetalactol, as well as with those of the Wiley Mass Spectral Database (Registry of Mass Spectral Data, Palisade Corporation, Newfield, NY, USA), peaks corresponding to the standards were identified (**Figure 5C**). The mass spectra of the compounds from the reaction mixtures, which correspond to 8-oxogeranial and *cis,trans*-nepetalactol, show fragmentation profiles characteristic for pure standards. GC/MS chromatograms of the control reaction showed only the peak corresponding to 8-oxogeranial. On the other hand, in the assays with *Nr*PRISE2 and *Nn*PRISE, in addition to 8-oxogeranial, peaks corresponding to *cis,trans*-nepetalactol were present (**Figures 5B, C**). This indicated the enzymatic conversion of 8-oxogeranial into nepetalactol in the presence of *Nr*PRISE and *Nn*PRISE.

An unambiguous assignment of the NMR spectra of *cis,trans*-nepetalactol and 8-oxogeranial was achieved using 1D (^1^H) and 2D COSY NMR techniques, and the results are shown in **Supplementary Figures 1** and **2**, respectively. NMR data resembled those reported in the literature (Liblikas et al., 2005). The obtained results of ^1^H NMR spectra of the reaction products with *Nn*PRISE show a clear appearance of peaks characteristic for *cis,trans*-nepetalactol (H-3, 6.01 and H-1, 4.85 ppm) and 8-oxogeranial (H-6, 6.42 and H-2, 5.91 ppm) which was unequivocal confirmed with crosspeaks in the COSY spectra. In the product of the reaction, which was catalyzed by the *Nr*PRISE2 enzyme, ^1^H NMR spectra indicate only the presence of *cis,trans*-nepetalactol peaks, while the concentration of 8-oxogeranial is below the detection level of the used spectroscopic technique (**Supplementary Figure 1**). Despite *Nn*PRISE being phylogenetically closer to P5βR, the NMR COSY spectrum confirmed that this recombinant enzyme converts 8-oxogeranial to *cis,trans*-nepetalactol, although incompletely (**Supplementary Figure 2**). In other words, *Nn*PRISE probably exhibits a weaker activity than *Nr*PRISE2 under the given reaction conditions, but kinetics studies are needed in order for this to be confirmed. It cannot be ruled out that altered reaction conditions (e.g. buffer pH value, MOPS concentration in the buffer, temperature, etc.) could lead to complete conversion and higher catalytic activity of *Nn*PRISE.

In *Nepeta* species ISYs perform the reduction of 8-oxogeranial and NEPSs are responsible for the subsequent cyclization (Lichman et al., 2019a and Lichman et al., 2019b). As both PRISEss (*Nr*PRISE2 and *Nn*PRISE) resulted in the production of *cis,trans*-nepetalactol in the absence of NEPSs, our results indicate that cyclization occurred spontaneously. This was also confirmed for *Nc*ISY2 and *Nm*ISY2 (Sherden et al., 2017), as well as for ISYs from plants of other genera (Geu-Flores et al., 2012; Alagna et al., 2016; Kries et al., 2017; Xiang et al., 2017; Fellows et al., 2018).

Moreover, in light of *Nr*PRISE2 demonstrated ISY activity both *in vitro* and likely *in planta*, in contrast to *Nn*PRISE which lacks suitable substrates for enzymatic action and exhibits a closer phylogenetic proximity to progesterone-5-β-reductases (P5BRs), we posit that a nomenclatural adjustment is warranted. Accordingly, we recommend the reclassification of *Nr*PRISE2 to *Nr*ISY, accurately reflecting its functional role as an iridoid synthase.

### Overall protein structure of *Nn*PRISE and *Nr*ISY

**Figure 6:**
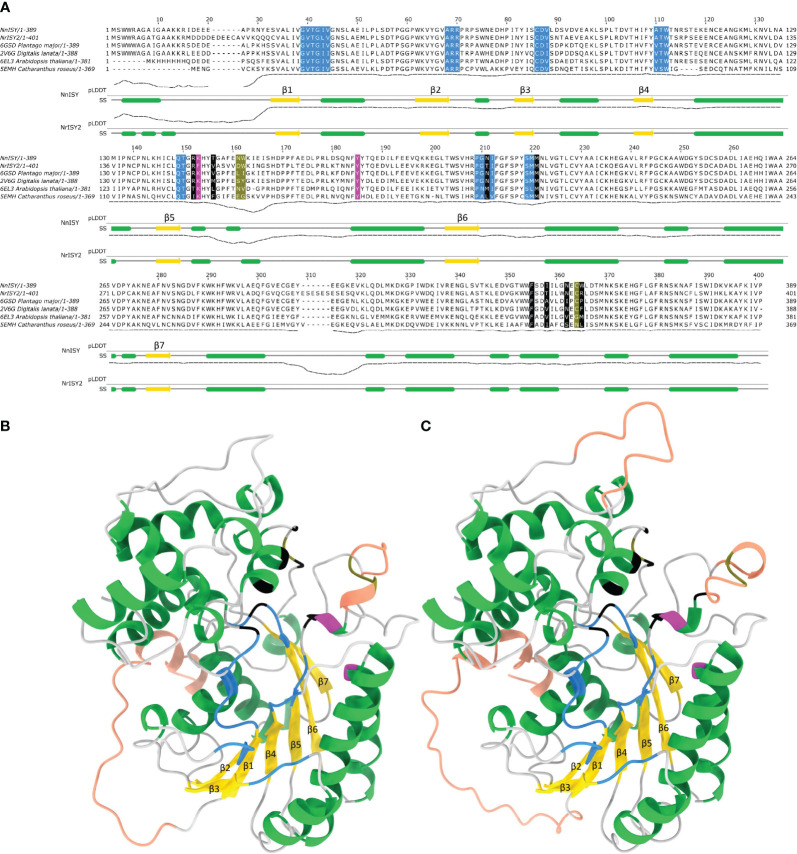
Sequence structure relationships in *Nn*PRISE and *Nr*ISY. **(A)** Multiple sequence alignment of *Nn*PRISE, *Nr*ISY and several ISY/P5βR enzymes with resolved 3D structures. Residues involved with NADP binding are colored blue (cdd: cd08948 annotation), catalytic residues are colored magenta (cdd: cd08948 annotation), residues forming the substrate binding cavity are colored black (Qin et al., 2016), while olive-colored residues are crucial loop residues determining substrate specificity (Sandholu et al., 2018). Under the alignment are the pLDDT score (predicted lDDT-C*α*) - a per-residue estimate of AlphaFold confidence on a scale from 0 – 100 and the secondary structure (SS) diagram based on the respective AlphaFold models: α-helices are in green, β-strands are in yellow. **(B)** *Nn*PRISE AlphaFold predicted structure, *α*-helices are colored green, *β*-strands yellow, coils grey, regions with pLDDT < 80 are shown in tangerine (potential disorder). Specific residues involved in cofactor and substrate binding, important loop residues and catalytic residues are colored as in **(A, C)** NrISY AlphaFold predicted structure: coloring is as in **(A, B)**.

In order to gain insights into the sequence structure relationships in *Nn*PRISE and *Nr*ISY, multiple sequence alignment with several ISY/5beta-POR enzymes with resolved 3D structures was performed (**Figure 6A**), and their structure was predicted using AlphaFold (**Figures 6B, C**). Overall, the structures resemble experimentally determined ISY and P5βR enzyme structures. After superposing with *Digitalis lanata* P5βR (PDB:2V6G, Thorn et al., 2008) the RMSD between aligned pairs of the backbone C-alpha atoms was 1.03 Å for *Nr*ISY and 0.86 Å for *Nn*PRISE. Both predicted structures have a high percent of Ramachandran favored angles (94 – 96%), few Ramachandran outliers, and low clash score (**Table 1**).

**Table 1:** Evaluation of AlphaFold predicted models of NnPRISE and NrISY.

Structure	MolProbity Score	Clash Score	Ramachandran Favored	Ramachandran Outliers	Rotamer Outliers	RMSD (2V6G)	TM (2V6G)	SI % (2V6G)
***Nn*PRISE**	1.2	1.8	96.12%	0.26%	0.00%	0.86	0.92	82%
***Nr*ISY**	1.32	1.76	94.24%	1.50%	0.00%	1.03	0.89	75%

The predicted structures contain a probably disordered N-terminal stretch of amino acids followed by a *β*-strand, which is a part of the parallel 7-stranded sheet sandwiched between alpha helices - the so called Rossmann fold (**Figures 6B, C**). The C-terminal extension region consists of alpha helices which are located between *β*6 and *β*7 and after *β*7. A short, most likely disordered (low pLDDT), loop is present between *β*5 and *β*6. This loop separates the two active site residues Lys and Tyr (Lys147 and Tyr 179 in *Nn*PRISE) which are located in the helical regions around it. Interestingly the Lys residue is replaced by Phe (Phe153) in *Nr*ISY. In the literature there is conflicting evidence about the role of the mentioned Lys residue; while it is conserved in ISY/P5βR, in *C. roseus* ISY the K146A, K146S, K146M and K146R mutants only suffered a slight loss of catalytic activities (Kries et al., 2015; Qin et al., 2016), while in the *Plantago major* P5βR multisubstrate oxido-reductase K147A and K147M mutations completely abrogated enzymatic activity (Fellows et al., 2018). Considering that *Nr*ISY is active, replacement of the mentioned Lys residue to Phe seems not to disturb the catalytic activity of this enzyme.

The NADP cofactor binding site in ISY/P5βR enzymes (**Figure 5A**; **Supplementary Figure 3**) is mostly made up from residues after the *β* strands of the Rossmann fold (**Supplementary Figure 3**). Due to the conservation of residues involved in interaction with NADP the hydrogen bonding network is similar between the *Nn*PRISE (**Supplementary Figure 3A**) and *Nr*ISY (**Supplementary Figure 3B**) predicted structures and the experimental *C. roseus* ISY (**Supplementary Figure 3C**) and *D. lanata* P5βR structures (**Supplementary Figure 3D**). In order to examine 8-oxogeranial binding to *Nn*PRISE and *Nr*ISY active sites docking was performed with 8-oxogeranial and different configurations of nepetalactol. 8-Oxogeranial is an *α,β* unsaturated aldehyde from both sides of the molecule (**Supplementary Figure 3A**). Due to conjugative stabilization the rotation around the *α,β* bond in *α,β* -unsaturated carbonyl compounds is constrained to s-cis and s-trans forms (Abraham et al., 2006; **Supplementary Figure 3A**) which exist in an equilibrium dictated by the bulkiness of the groups on the carbonyl, C*α* and C*β* atoms. Interestingly 8-oxogeranial is currently present in three enzyme structures deposited to the PDB, two from *C. roseus ISY* - 5COB (Qin et al., 2016) and 5DBI (Hu et al., 2015), as well as one from *P. major* - 5MLH (Fellows et al., 2018). In two of these structures, 5COB and 5MLH, the C1-C2 *α,β* bond is neither in the s-trans, nor the s-cis form but is rather in between these two forms (**Supplementary Figure 3B**). It is unclear if this is due to poor goodness of fit of the ligand to experimental data (8 percentile for 5COB, 13% 5MLH and 75% 5DBI) or some other factor. On the other hand, the substrate in the 5DBI structure is in the 1,2 s-trans, 7,8 s-cis conformation, and Hu and associates (2015) provide a mechanistical explanation how this 8-oxogeranialtransoid (1,2 s-trans) is converted to a shunt-reaction product S-10-oxocitronellal (8-oxocitronellal) while the cisoid (1,2 s-cis), as yet experimentally unobserved, is converted to nepetalactol. Some 8-oxogeranial *α,β* conformations are more stable than others as revealed by examining their energy using several forcefields (**Supplemetary Table 4**).

**Figure 7:**
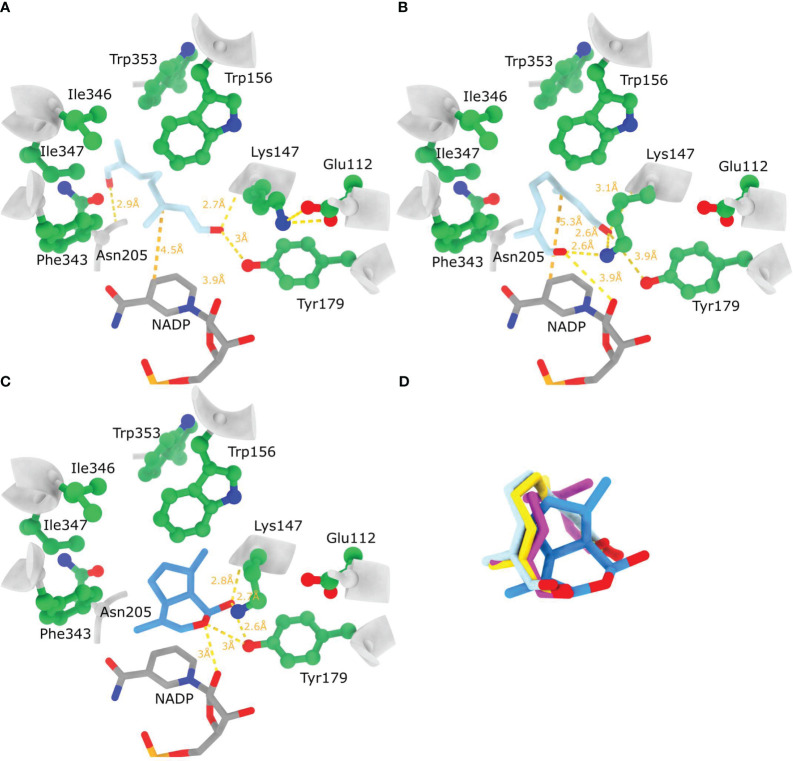
Substrate and product binding to the AlphaFold model of *Nn*PRISE. **(A)** Highest affinity docked position of 1,2 s-*trans*, 7,8 s-*cis* 8-oxogeranial to the rigid *Nn*PRISE active site. **(B)** Highest affinity docked position of 1,2 s-*trans*, 7,8 s-*cis* 8-oxogeranial to the *Nn*PRISE active site where the side chains of Lys147 and Tyr179 were allowed to be flexible. **(C)** Highest affinity docked position of 1S- *cis*,*trans* nepetalactol (PubChem: 11286692) to NnISY active site where the side chains of Lys147 and Tyr179 were allowed to be flexible. **(D)** Superposition of bound substrate, hydride addition products [*trans*- 8-oxocitronellyl enolate (yellow) and *cis*- 8-oxocitronellyl enolate (magenta)] and cyclisation product. Hydrogen bonding between the docked ligands and protein residues is shown with dashed yellow lines (relaxed hydrogen bonding criteria - distance tolerance 1 Å and angle tolerance 20°).

In view of all this, docking was performed with the four 8-oxogeranial conformational variants (**Supplementary Table 4**) so that the rotation of *α,β* bonds was not permitted. When performing docking to the rigid *Nn*PRISE model active site the highest affinity docked position was of 1,2 s-trans, 7,8 s-cis 8-oxogeranial (same conformer as observed in 5DBI) which assumed an extended conformation where the C1 carbonyl oxygen is at hydrogen bond distance from Tyr179 and the backbone N of Lys147, while the C8 carbonyl oxygen is proximate to the backbone N of Asn205 (**Figure 7A**). The docked ligand is in a similar position as in 5DBI regarding the hydrogen bonding of C1 carbonyl oxygen as well as the positioning of the C2=C3 double bond on top of the NADP cofactor. In this structure the *ϵ*-NH_3_ group of Lys147 appears not to have a role in ligand binding because it is facing away from the ligand, and is H-bonded to Glu112 (**Figure 7A**). Contrary to this when flexible docking was employed (Lys147 and Tyr179 side chains) 1,2 s-trans, 7,8 s-cis 8-oxogeranial assumed a contorted conformation with both carbonyl groups in the direction of the active site residues Lys147 and Tyr179, just above the nicotinamide ring of NADP (**Figure 7B**). The ϵ-NH_3_ group of Lys147 has moved away from Glu112 to contribute to H-bonding with both substrate carbonyl groups (~2.6 Å, **Figure 7A**). The carbonyl oxygen on C8 is positioned less than 4 Å away from Tyr179 Oζ, while the carbonyl oxygen on C1 is less than 4 Å away from the 2’ ribose OH group. This docked position suggests 8-oxogeranial binding to the active site in a conformation that could promote cyclisation. In addition to this the products of the hydride addition reaction trans- and cis- 8-oxocitronellyl enolates assume a very similar highest scoring docked position (**Figure 7D**) to the before mentioned 1,2 s-trans, 7,8 s-cis 8-oxogeranial. When nepetalactol (1S cis,trans) was docked, Lys147 has pooled towards Tyr179 reducing the distances of nepetalactol oxygens to Tyr179 Oζ, Lys147 ε-NH3 and 2’ ribose OH group (**Figure 7C**). Taken together these *in silico* results suggest that if the Lys147 ε-NH_3_ group remains H-bonded to Glu112 during 8-oxogeranial binding then the enzyme most likely catalyzes just the reduction reaction and not the subsequent cyclisation. Lichman et al. (2019a) proposed that ISY in *Nepeta* performs just the reduction of 8-oxogeranial to 8-oxocitronellyl enol/enolate which is converted to nepetalactol either by spontaneous cyclization in solution, or by cyclisation catalyzed by Nepetalactol-related short-chain reductases (NEPS) which subsequently catalyze the reduction of nepetalactol to nepetalactone. In addition, different NEPS isoforms promote formation of different nepetalactone stereochemical configurations (Lichman et al., 2019a).

**Figure 8:**
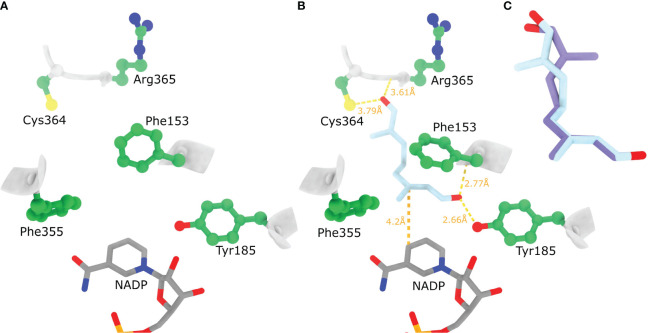
Ligand binding to the AlphaFold model of *Nr*ISY. **(A)** AlphaFold model of *Nr*ISY active site. **(B)** Highest affinity docked position of 1,2 s-*trans*, 7,8 s-*trans* 8-oxogeranial to *Nr*ISY active site where the side chains of Phe153 and Tyr185were allowed to be flexible. **(C)** Superposition of *Nr*ISY docked 1,2 s-*trans*, 7,8 s-*trans* 8-oxogeranial (light blue) and 1,2 s-*trans*, 7,8 s-*cis* 8-oxogeranial (purple). Hydrogen bonding between the docked ligands and protein residues is shown with dashed yellow lines (relaxed hydrogen bonding criteria - distance tolerance 1 Å and angle tolerance 20°).

When docking different conformations of 8-oxogeranial to the rigid active site of the *Nr*ISY model the resulting docked positions were not compatible with the catalysis of the hydride attack because the substrate conformations were bound away from NADP and Tyr185 (not shown). Presumably because the side chain of Phe153 is oriented in such a way that prohibits the 8OG C2=C3 double bound to orient on top of NADP cofactor (**Figure 8A**). Indeed, with the flexible docking procedure the Phe153 aromatic ring rotated ~90 degrees allowing 8-oxogeranial to bind so that the C1 carbonyl oxygen is at hydrogen bond distance from Tyr185 and the backbone N of Phe153, while the double bond prone to reduction is sandwiched between Phe153 and the nicotinamide ring (**Figure 8B**). Based on the docked poses it seems *Nr*ISY does not bind 8-oxogeranial in a conformation suitable for cyclization in the enzyme active site. The main difference between *Nn*PRISE and *Nr*ISY is that the active site Lys147 is replaced by Phe (Phe153 in NrISY), and that the *Nn*PRISE substrate binding site is more spatially confined with bulky hydrophobic amino acids such as Trp353 and Trp156. In *Nr*ISY Trp353_NnPRISE_ is replaced by Arg365, while Trp162 which is by position in the sequence equivalent to Trp156_NnPRISE_ is positioned away from the substrate binding cleft. Superposition of NrISY docked 1,2 s-trans, 7,8 s-trans 8-oxogeranial (light blue) and 1,2 s-trans, 7,8 s-cis 8-oxogeranial (purple) is presented in **Figure 8C**.

## Conclusions

Although several ISYs were previously characterized from *Nepeta* species, this is, to the best of our knowledge, the first record of functional ISY (*Nr*ISY) in *trans*,*cis-nepetalactone* and 5,9-dehydronepetalactone predominating *N. rtanjensis*. Displaying high catalytic activity under *in vitro* conditions, *Nr*ISY is an enzyme showing organ-specific expression profiles in plants, with leaves being enriched with the enzyme transcripts. On the other hand, PRISE isolated from leaves of *N. nervosa* (*Nn*PRISE) shares high level of similarity with the *Nr*ISY, but is characterized by more spatially confined substrate binding site, which is, most likely, responsible for less efficient conversion of 8-oxogearnial to *cis,trans*-nepetalactol. Future kinetics experiments will be aimed to confirm these statements. Described *Nn*PRISE from *N. nervosa* is actually phylogenetically closer to the representatives of the Family 1 isoforms, designated as P5βRs and displaying very low catalytic activity. However, based on the functional genomics and bioinformatics (3D modeling and molecular docking analyses) data, it could be presumed that this enzyme displays ISY-like function under *in vitro* conditions. Although *Nn*PRISE is expressed in high levels in stems and leaves of *N. nervosa*, more studies are needed to confirm its activity *in vivo*.

MeJA- induced accumulation of iridoids in *N. rtanjensis* leaves was followed by the elevated co-expression of the majority of the iridoid biosynthesis-related genes and transcription factors, which indicated transcriptional regulation of their activity. The same treatment with MeJA slightly increased the expression levels of the candidate biosynthetic genes in *N. nervosa* leaves; however, this induced no appearance of iridoid glycosides and iridoid aglycones (nepetalactone, its precursors, or derivatives). Although *Nepeta nervosa* is lacking detectable amounts of iridoids in leaves, even under MeJA-elicitation conditions, this species most likely possesses iridoid biosynthetic platform, which is silenced. Based on the obtained metabolomics and transcriptomics data, we could presume that there are a few missing points in the iridoid biosynthetic platform of *N. nervosa*, which disables the biosynthesis of iridoids: 1) the absence of GES − like activity, due to the gene silencing, which results in the absence of both iridoid aglycones and iridoid glycosides; 2) the activity of some other NEPSs and MLPLs besides *Nn*NEPS1 and *Nn*MLPL are essential for iridoid aglycones biosynthesis. In the quest for answers, our ongoing work is conducted towards testing these robust hypotheses and re-establishing the biosynthesis of iridoids in this remarkable plant. Interestingly, the majority of putative iridoid biosynthesis-related gens are expressed in leaves of *N. nervosa*, even if there are no substrates to act upon, which is an interesting paradox. The question arises as to how energetically profitable it is for plants to express enzymes that are unable to fulfill their function within specific biosynthetic routes of specialized metabolites. Most likely this is not an isolated example within the plant kingdom, but it certainly deserves our attention.

## Data availability statement

The datasets presented in this study can be found in online repositories. The names of the repository/repositories and accession number(s) can be found in the article/**Supplementary Material**.

## Author contributions

DMi, DM, and NA conceived and designed the experiments. NA, DM, UG, JN, SD, JB, MM, MD, BA, LP, and DMi performed the experiments. NA, DM, UG, and DMi organized and wrote the manuscript with editing from all the authors. All authors contributed to the article and approved the submitted version.
