# Supplementary material for: Functional iridoid synthases from iridoid producing and non-producing Nepeta species (subfam. Nepetoidae, fam. Lamiaceae)
Source: Front Plant Sci. 2024 Jan 3;14:1211453. doi: 10.3389/fpls.2023.1211453 (PMC10792066; doi:10.3389/fpls.2023.1211453)
Supplement: Supplementary file 1 [file Presentation_1.pptx]

## Slide 1
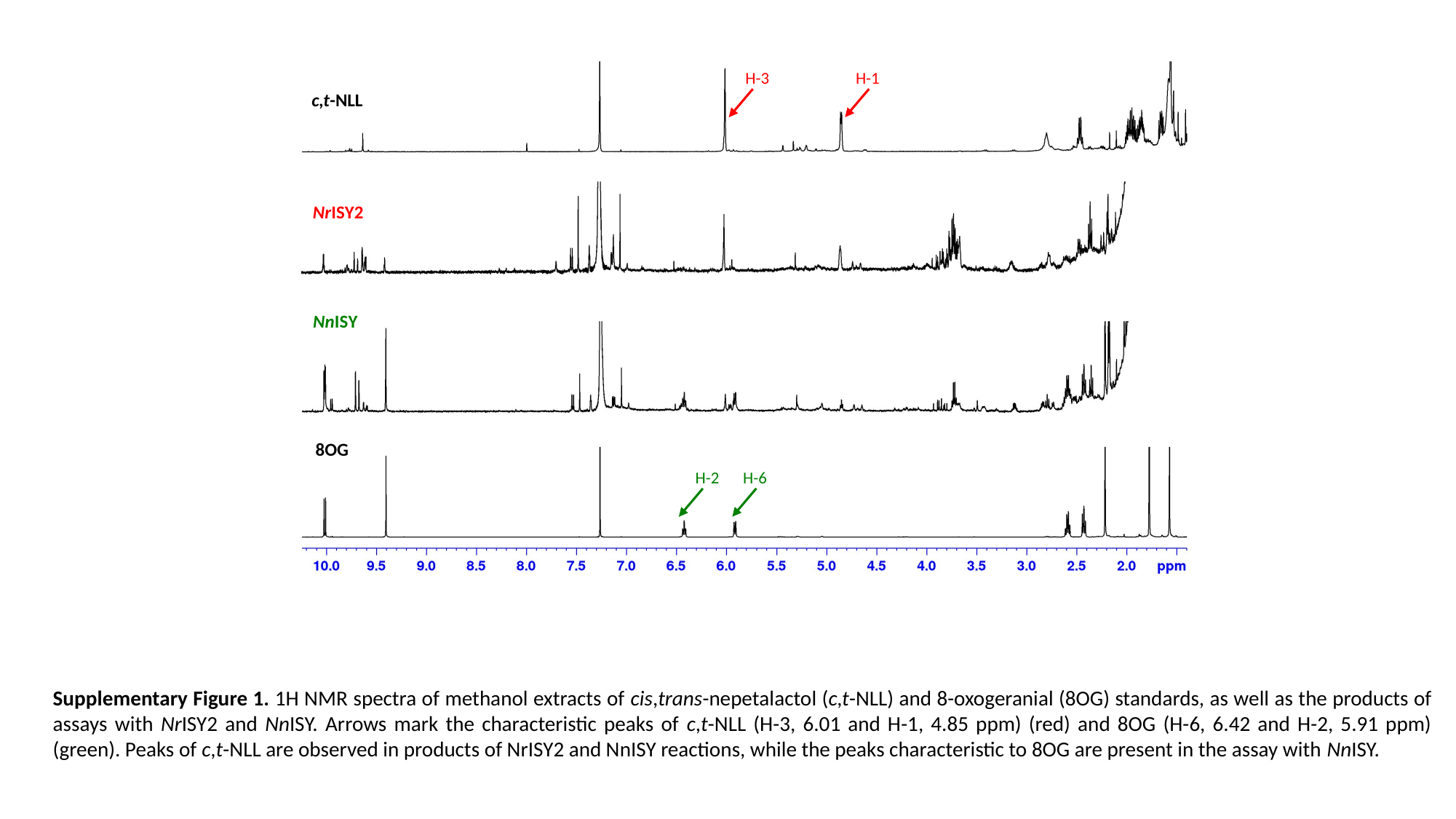

H-3
H-1
c,t-NLL
NrISY2
NnISY
8OG
H-2
H-6
Supplementary Figure 1. 1H NMR spectra of methanol extracts of cis,trans-nepetalactol (c,t-NLL) and 8-oxogeranial (8OG) standards, as well as the products of assays with NrISY2 and NnISY. Arrows mark the characteristic peaks of c,t-NLL (H-3, 6.01 and H-1, 4.85 ppm) (red) and 8OG (H-6, 6.42 and H-2, 5.91 ppm) (green). Peaks of c,t-NLL are observed in products of NrISY2 and NnISY reactions, while the peaks characteristic to 8OG are present in the assay with NnISY.

## Slide 2
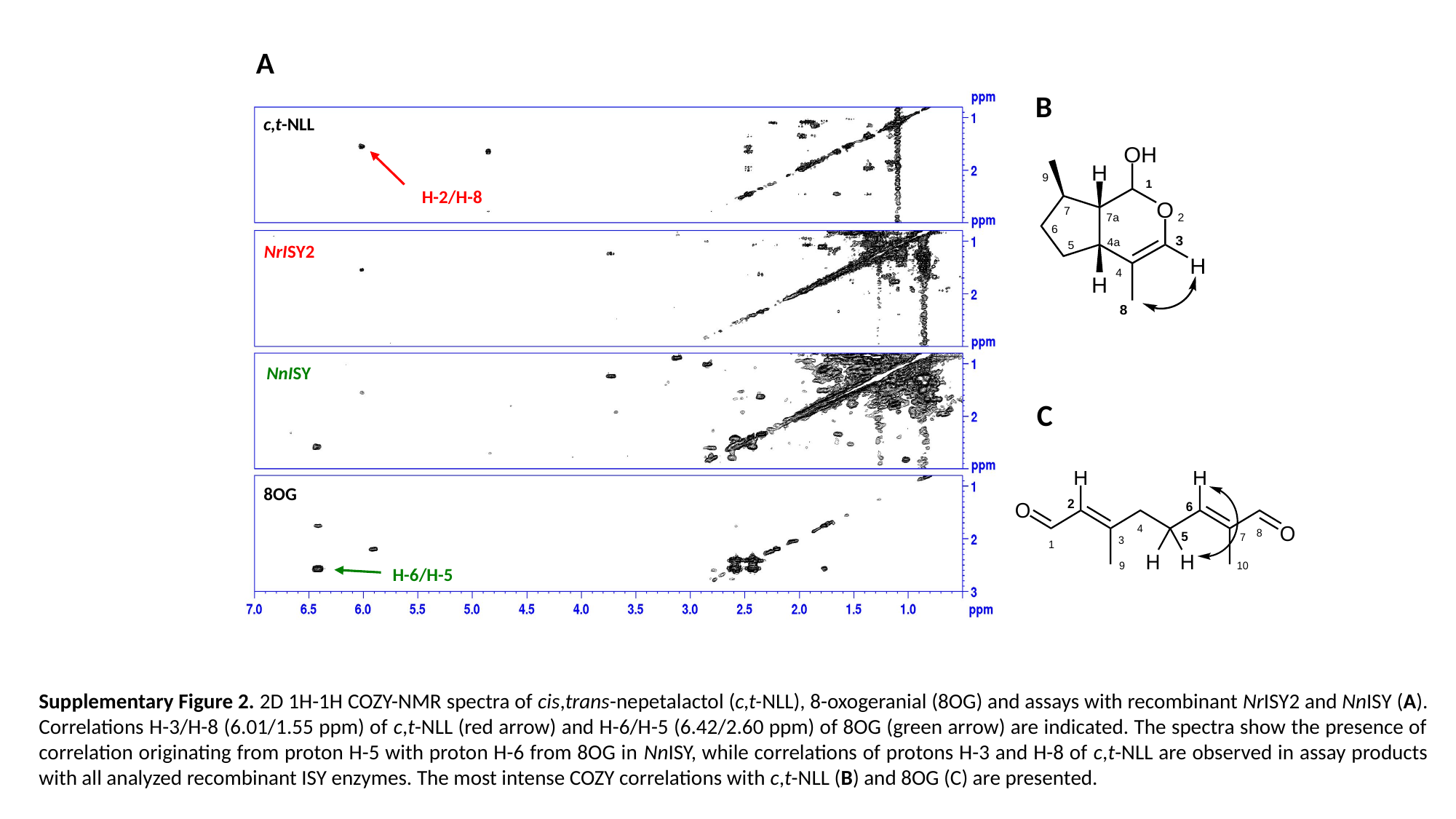

A
B
c,t-NLL
H-2/H-8
NrISY2
NnISY
8OG
H-6/H-5
C
Supplementary Figure 2. 2D 1H-1H COZY-NMR spectra of cis,trans-nepetalactol (c,t-NLL), 8-oxogeranial (8OG) and assays with recombinant NrISY2 and NnISY (A). Correlations H-3/H-8 (6.01/1.55 ppm) of c,t-NLL (red arrow) and H-6/H-5 (6.42/2.60 ppm) of 8OG (green arrow) are indicated. The spectra show the presence of correlation originating from proton H-5 with proton H-6 from 8OG in NnISY, while correlations of protons H-3 and H-8 of c,t-NLL are observed in assay products with all analyzed recombinant ISY enzymes. The most intense COZY correlations with c,t-NLL (B) and 8OG (C) are presented.

## Slide 3
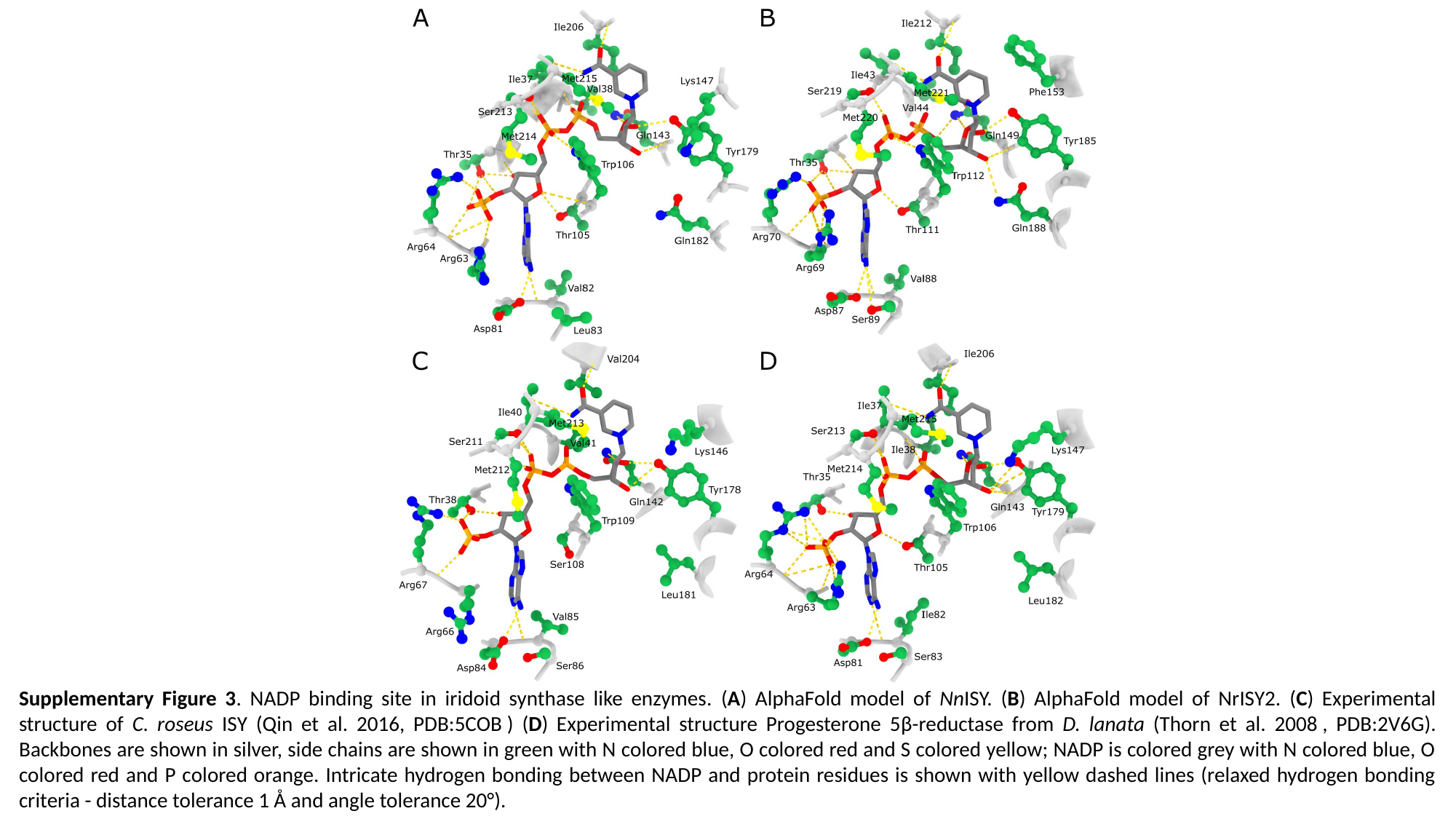

Supplementary Figure 3. NADP binding site in iridoid synthase like enzymes. (A) AlphaFold model of NnISY. (B) AlphaFold model of NrISY2. (C) Experimental structure of C. roseus ISY (Qin et al. 2016, PDB:5COB ) (D) Experimental structure Progesterone 5β-reductase from D. lanata (Thorn et al. 2008 , PDB:2V6G). Backbones are shown in silver, side chains are shown in green with N colored blue, O colored red and S colored yellow; NADP is colored grey with N colored blue, O colored red and P colored orange. Intricate hydrogen bonding between NADP and protein residues is shown with yellow dashed lines (relaxed hydrogen bonding criteria - distance tolerance 1 Å and angle tolerance 20°).

## Slide 4
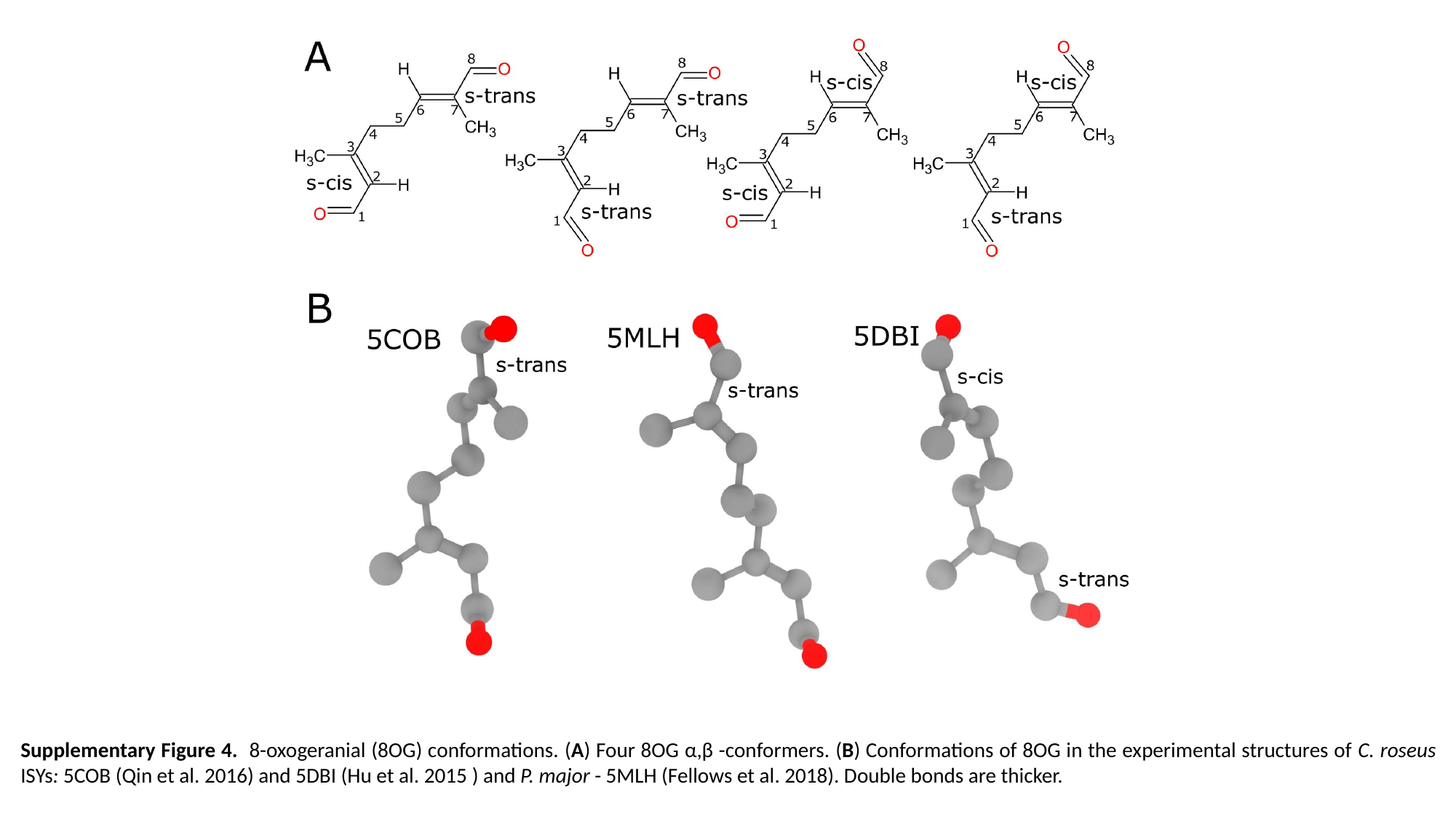

Supplementary Figure 4. 8-oxogeranial (8OG) conformations. (A) Four 8OG α,β -conformers. (B) Conformations of 8OG in the experimental structures of C. roseus ISYs: 5COB (Qin et al. 2016) and 5DBI (Hu et al. 2015 ) and P. major - 5MLH (Fellows et al. 2018). Double bonds are thicker.

## Slide 5
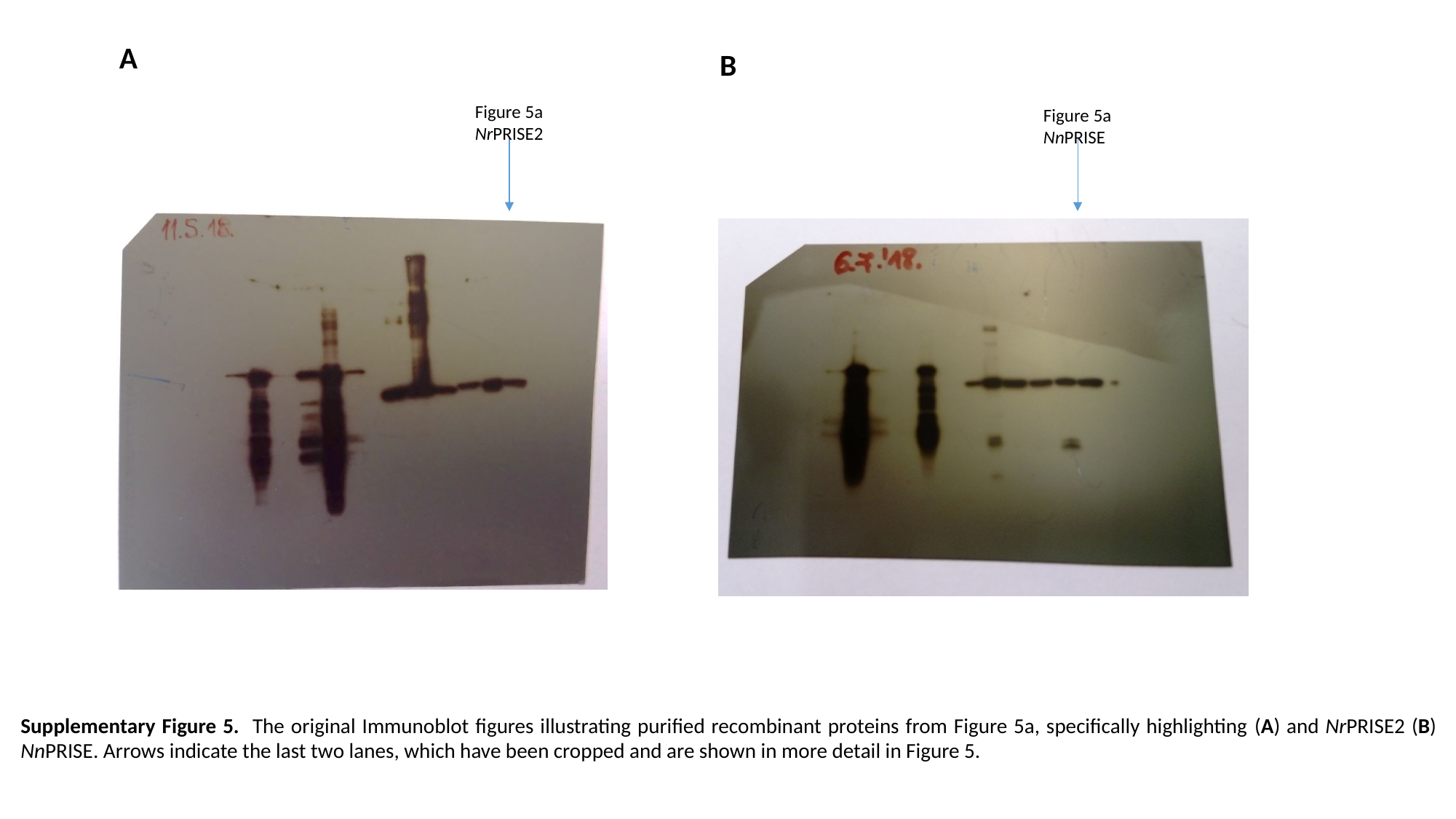

A
B
Figure 5a
NrPRISE2
Figure 5a
NnPRISE
Supplementary Figure 5. The original Immunoblot figures illustrating purified recombinant proteins from Figure 5a, specifically highlighting (A) and NrPRISE2 (B) NnPRISE. Arrows indicate the last two lanes, which have been cropped and are shown in more detail in Figure 5.
