# Supplementary material for: Functional iridoid synthases from iridoid producing and non-producing Nepeta species (subfam. Nepetoidae, fam. Lamiaceae)
Source: Front Plant Sci. 2024 Jan 3;14:1211453. doi: 10.3389/fpls.2023.1211453 (PMC10792066; doi:10.3389/fpls.2023.1211453)
Supplement: Supplementary file 2 [file Table_1.docx]

**Supplementary Table 1. Accession numbers of sequences and primer combinations used for full-length ISY/PRISE gene amplification and qPCR analysis of nepetalactone biosynthetic genes and the corresponding transcription factors in *N. rtanjensis* and *N. nervosa*.**

| **Sequence abbreviation** | **Accession number** | **Primer name** | **Primer sequence (5’-3’)** | **Species** | **Target gene** |
| --- | --- | --- | --- | --- | --- |
| NrISY/ | **MN936115**  **OQ858852** | IrSyn_FL_F  IrSyn_FL_R1  IrSyn_FL_R2 | ATGAGCTGGWGGGCT | *N. rtanjensis/*  *N.nervosa* | ISY |
| NnISY |  |  | TCAAGGAACGATCTTGTAAGC |  |  |
|  |  |  | TCAGGGAACAATCTTGAAAGC |  |  |
| NrISY/ | **MN936115**  **OQ858852** | IrSyn_FL_F_SacI | GCATGAGAGCTCATGAGCTGGTGGTG | *N. rtanjensis/*  *N.nervosa* | ISY |
| NnISY |  | IrSyn_FL_R1_KpnI | GCATGAGGTACCTCAGGGAACAATCTTGAAAGC |  |  |
|  |  | IrSyn_FL_R2_KpnI | GCATGAGGTACCTCAAGGAACGATCTTGTAAGC |  |  |
| NrGAPDH | **MN936120** | NrGAPDH F  NrGAPDH R | GGCAAAGTTCTCCCTGCTCT  ATACTCGACCTGTTGTCGCC | *N. rtanjensis/*  *N.nervosa* | GAPDH |
| NrGPPS | **MN936110** | NrGPPS F | GACCCAATGCTGTCGATTTT | *N. rtanjensis* | GPPS |
|  |  | NrGPPS R | GCAAGTAGGCCTCCACAGAC |  |  |
| NrGES | **MN936111**  **MN936111**  **MN936111**  **MN936111**  **MN936111** | NrGES F | TGGTTCAATGGTGGAATTGC | *N. rtanjensis* | GES |
|  |  | NrGES R | GAGATCATCCCAAAGGCGAA |  |  |
| NrG8H | **MN936112** | NrG8H F | CGTCGCTAAAGAAGTCTTCCA | *N. rtanjensis* | G8H |
|  |  | NrG8H R | CGGAGTACTTGAACTGGTCGT |  |  |
| Nr8HGO | **MN936113** | Nr8HGO F | TGGTTAAGGTGGATCCGAAG | *N. rtanjensis* | 8HGO |
|  |  | Nr8HGO R | CTTTTGCCTTATCGCGTCTC |  |  |
| NrPRISE1 | **MN936114** | NrISY1 F | TGGGGAGTATGAGGAAGGGC | *N. rtanjensis* | PRISE1 |
|  |  | NrISY1 R | AACCACCAATTCCCCACATC |  |  |
| NrISY2 | **MN936115** | NrISY2 F | ATGTGGGGAGTACGAGTCCG | *N. rtanjensis* | ISY2 |
|  |  | NrISY2 R | GGTCCGGTGTGGGATCAAAT |  |  |
| NrNEPS1 | **MN936116** | NrNEPS1 F | AACTGCGTGACACCATCTGT | *N. rtanjensis* | NEPS1 |
|  |  | NrNEPS1 R | ATGTCCAGTGATGAAGGCGG |  |  |
| NrNEPS2 | **MN936118** | NrNEPS2 F | ATGAGCCAATCTGAGTCCGC | *N. rtanjensis* | NEPS2 |
|  |  | NrNEPS2 R | ATCAATGTATCCGCCCCTCG |  |  |
| NrNEPS3 | **MN936119** | NrNEPS3 F | GGCTAACAATTCAGTGATGATG | *N. rtanjensis* | NEPS3 |
|  |  | NrNEPS3 R | CCGTACCTCACGAACATGC |  |  |
| NrNEPS4 | **MN936117** | NrNEPS4 F | CGACAGCATCAAAGGGAGGA AAG | *N. rtanjensis* | NEPS4 |
|  |  | NrNEPS4 R | ACCAAGGGCGACACACAG |  |  |
| NrCOI1 | **MN936121** | NrCOI1 F | TCCTCTATGCTCTGCTTGATACTG | *N. rtanjensis/*  *N.nervosa* | COI1 |
|  |  | NrCOI1 R | TCGTGTTCATCGGCTCCAC | *N.nervosa* |  |
| NrJAZ3 | **MN936122** | NrJAZ3 F | GTTCATTTGTCGCCTCCTATCG | *N. rtanjensis/*  *N.nervosa* | YAZ3 |
|  |  | NrJAZ3 R | ACCCTCTCCTTACGCTTCTC | *N.nervosa* |  |
| NrMYC2 | **MN936124** | NrMYC2 F | ATTCATTACCTCCCGCTTGC | *N. rtanjensis/*  *N.nervosa* | MYC2 |
|  |  | NrMYC2 R | CTTCCGCTCTCTGGCTCAC | *N.nervosa* |  |
| NrYABBY5 | **MN936125** | NrYABBY5 F | GATGTGGCGGAGCAGATGT | *N. rtanjensis/*  *N.nervosa* | YABBY5 |
|  |  | NrYABBY5 R | AGTCCAGAGATGGGAGCAGT | *N.nervosa* |  |
|  |  |  |  |  |  |
| NnGPPS | **OQ858849** | NnGPPS F | CCGGTCAGTTTCTCGACCTC | *N.nervosa* | GPPS |
|  |  | NnGPPS R | GGCTCGCCCATATTTCCTCA |  |  |
| NnG8H | **OQ858850** | NnG8H F | CGTCGCCAAAGAAGTCCTCCA | *N.nervosa* | G8H |
|  |  | NnG8H R | CGGAGTACTTGAACTGGTCGT |  |  |
| Nn8HGO | **OQ858851** | Nn8HGO F | ACCGGAGGAGTAGGTGTGGA | *N.nervosa* | 8HGO |
|  |  | Nn8HGO R | TCCCCAACAGCAGAGGAACG |  |  |
| NnPRISE | **OQ858852** | NnISY F | CATATTCTATGCCACATGGACC | *N.nervosa* | PRISE |
|  |  | NnISY R | TGAACGGAGGATCGTGACTTC |  |  |
| NnNEPS1 | **OQ858853** | NnNEPS1 F | GTGGCGGAGTCCATCGGTAA | *N.nervosa* | NEPS1 |
|  |  | NnNEPS1 R | GGTCGAGGTCCATCACGGTT |  |  |
