# Supplementary material for: Functional iridoid synthases from iridoid producing and non-producing Nepeta species (subfam. Nepetoidae, fam. Lamiaceae)
Source: Front Plant Sci. 2024 Jan 3;14:1211453. doi: 10.3389/fpls.2023.1211453 (PMC10792066; doi:10.3389/fpls.2023.1211453)
Supplement: Supplementary file 3 [file Table_2.docx]

**Supplementary Table 2**. GC/MS characterization of methanol extracts of *N. rtanjensis* and *N. nervosa* leaves from *in vitro* grown plants (relative content, %).

| **No** | **Component RT** | **Assignment** | **Molecular formula** | ***N. rtanjensis*** | ***N. nervosa*** |
| --- | --- | --- | --- | --- | --- |
| 1 | 6.40 | ***α*-Thujene** | C10H16 | 1.79 | 0.12 |
| 2 | 6.87 | ***α*-Pinene** | C10H16 | 0.22 | 0.89 |
| 3 | 6.98 | **β-Myrcene** | C10H16 | 0.05 |  |
| 4 | 7.42 | **Eucalyptol** | C10H18O | 0.07 |  |
| 5 | 8.79 | **Terpinyl formate** | C11H18O2 | 0.03 |  |
| 6 | 9.10 | **2,3-Epoxy-geranyl acetate** | C12H20O3 | 0.23 |  |
| 7 | 9.23 | ***trans*-*p*-Menth-2-en-7-ol** | C10H18O | 0.31 |  |
| 8 | 9.90 | ***β*-Selinene** | C11H20O | 0.08 |  |
| 9 | 10.04 | ***p*-Menth-4-en-3-one** | C10H16O | 0.07 |  |
| 10 | 10.09 | ***cis*,*trans*-Nepetalactone** | C10H14O2 | 1.80 |  |
| 11 | 10.12 | **Thymol** | C10H14O | 0.02 |  |
| 12 | 10.19 | ***α*-Copaene** | C15H24 | 1.73 |  |
| 13 | 10.31 | ***trans*,*cis*-Nepetalactone** | C10H14O2 | 17.81 |  |
| 14 | 10.62 | **(E)-*β*-Farnesene** | C15H24 | 0.98 | 0.64 |
| 15 | 10.72 | **1,7,7-Trimethyl-5-oxo-norbornan-2-yl acetate** | C12H18O3 | 2.77 |  |
| 16 | 10.79 | **(+)-*epi*-Bicyclosesquiphellandrene** | C15H24 | 0.73 |  |
| 17 | 10.86 | **1,8-Methanonaphthalen** | C11H8 | 0.02 |  |
| 18 | 10.91 | **Germacrene D** | C15H24 | 4.71 | 23.89 |
| 19 | 11.01 | ***p*-Mentha-2,4(8)-diene** | C10H16 | 0.03 |  |
| 20 | 11.10 | **5,9-Dehydronepetalactone** | C10H12O2 | 24.25 |  |
| 21 | 11.23 | **γ-Cadinene** | C15H24 | 2.70 | 0.30 |
| 22 | 11.49 | **Germacrene D-4-ol** | C15H26O | 0.24 | 4.51 |
| 23 | 11.79 | **10-*Epi*-gamma-eudesmol** | C15H26O | 0.05 |  |
| 24 | 12.82 | **Neophytadiene** | C20H38 | 0.20 | 1.45 |
| 25 | 14.19 | **Phytol** | C20H40O | 1.23 | 6.87 |
| 26 | 15.60 | **2,2'-Methylenebis-(6-tert-butyl)-*p*-cresol** | C23H32O2 | 0.12 | 0.57 |
| 27 | 15.80 | ***p*-Menth-8-en-3-ol acetate** | C12H20O2 | 0.29 |  |
| 28 | 17.31 | **Squalene** | C30H50 |  | 0.72 |
