# Supplementary material for: Functional iridoid synthases from iridoid producing and non-producing Nepeta species (subfam. Nepetoidae, fam. Lamiaceae)
Source: Front Plant Sci. 2024 Jan 3;14:1211453. doi: 10.3389/fpls.2023.1211453 (PMC10792066; doi:10.3389/fpls.2023.1211453)
Supplement: Supplementary file 4 [file Table_3.docx]

**Supplementary Table 3**. qToF MS data of methanol extracts of leaves of *in vitro* grown *Nepeta nervosa* and *N. rtanjensis*.

| **No** | **Compound names** | ***t*_R_, min** | **Molecular formula** | **Calculated mass, *m/z*** | **Exact mass, *m/z*** | **mDa** | **MS^2^ Fragments, *m/z* (% Base Peak)** | ***NR*** | ***NN*** | **References** |
| --- | --- | --- | --- | --- | --- | --- | --- | --- | --- | --- |
| ***Hydroxycinnamic acids*** | | | | | | | | | | |
| **1** | **3-*O*-Caffeoylquinic acid*^a^*** | 2.02 | C_16_H_17_O_9_^–^ | 353.08781 | 353.08237 | 5.44 | 135.03392(81), 161.01247(5), 179.02343(44), **191.04586**(100) | – | ✚ | Mišić et al., 2015 |
| **2** | **5-*O*-Caffeoylquinic acid*^a^*** | 3.23 | C_16_H_17_O_9_^–^ | 353.08781 | 353.08225 | 5.56 | 127.02164(3), 161.01605(3), **191.04622**(100), 192.04590(11) | – | ✚ | Mišić et al., 2015 |
| **3** | **5-*O*-Caffeoylquinic acid isomer*^a^*** | 3.97 | C_16_H_17_O_9_^–^ | 353.08781 | 353.08271 | 5.09 | **191.04531**(100), 192.04970(9) | – | ✚ | Mišić et al., 2015 |
| **4** | **Caffeic acid*^a^*** | 4.44 | C_9_H_7_O_4_^–^ | 179.03440 | 179.03087 | 3.53 | 107.03872(10), 117.02291(7), 134.02565(68), **135.03371**(100) | ✚ | – | Aničić et al., 2021 |
| **5** | **Feruloylquinic acid hexoside*^c^*** | 4.85 | C_23_H_29_O_14_^–^ | 529.15628 | 529.15437 | 1.91 | 173.03593(43), **191.04605**(100), 193.03836(32), 367.09573(11) | – | ✚ | Sun et al., 2015 |
| **6** | **Feruloylquinic acid*^a^*** | 5.52 | C_17_H_19_O_9_^–^ | 367.10294 | 367.10087 | 2.07 | 111.03427(13), 134.02578(30), 173.03449(12), **191.04587**(100), 193.04078(17) | ✚ | ✚ | Aničić et al., 2021 |
| **7** | **Rosmarinic acid*^a^*** | 5.82 | C_18_H_15_O_8_^–^ | 359.07724 | 359.07453 | 2.71 | 109.05026(9), **135.07008**(100), 153.08040(25), 197.07058(6) | ✚ | ✚ | Aničić et al., 2021 |
| **8** | **Ferulic acid pentoside*^c^*** | 6.19 | C_15_H_17_O_8_^–^ | 325.09289 | 325.09008 | 2.81 | **134.02598**(100), 135.02516(10), 149.04218(6), 193.04075(10) | – | ✚ | Bauer et al., 2012 |
| **9** | **Ferulic acid*^a^*** | 7.14 | C_10_H_9_O_4_^–^ | 193.05010 | 193.04505 | 5.05 | 107.03413(12), **133.01944**(100), 134.01899(13), 161.00761(45) | ✚ | – | Mišić et al., 2015 |
| **10** | **Nepetoidin A or B*^a^*** | 10.64 | C_17_H_13_O_6_^–^ | 313.07176 | 313.06694 | 4.82 | 151.02897(8), **161.01356**(100), 179.02313(5), 267.05859(12) | ✚ | – | Aničić et al., 2021 |
| ***Flavonoids*** | | | | | | | | | | |
| **11** | **Luteolin 7-*O*-dihexuronide*^a^*** | 5.59 | C_27_H_25_O_18_^–^ | 637.10464 | 637.10596 | -1.32 | 175.01490(6), 193.02476(22), **285.03234**(100), 351.05123(70) | ✚ | – | Dienaitė et al., 2018 |
| **12** | **Hydroxy-trimethoxyflavone malonyl-hexoside*^c^*** | 8.38 | C_27_H_29_O_14_^+^ | 577.15518 | 577.15375 | 1.43 | **329.10012**(100), 330.10347(15), 331.10558(4), 313.06877(3), 109.02683(3) | ✚ | – | NA |
| **13** | **Cirsimaritin*^a^*** | 10.00 | C_17_H_13_O_6_^–^ | 313.07120 | 313.06693 | 4.27 | 227.02733(7), 255.02172(14), **283.01789**(100), 297.03105(8) | ✚ | – | Aničić et al., 2021 |
| **14** | **Acacetin*^a^*** | 10.44 | C_16_H_13_O_5_^+^ | 285.07575 | 285.07717 | -1.42 | 124.01556(11), 167.03223(21), **242.05556**(100), 270.05046(53) | ✚ | – | Kashchenko & Olennikov, 2016 |
| ***Iridoid glycosides*** | | | | | | | | | | |
| **15** | **5-Deoxylamiol*^b^*** | 4.58 | C_16_H_25_O_9_^–^ | 361.14993 | 361.14545 | 4.48 | **101.01003**(100), 113.00938(71), 119.07332(64), 135.06881(15), 137.08517(99), 181.0707(14) | ✚ | – | Alipieva et al., 2007 |
| **16** | **Geniposide*^a^*** | 4.78 | C_17_H_23_O_10_^–^ | 387.12967 | 387.12754 | 2.13 | 101.01173(10), **161.04958**(100) | ✚ | – | Aničić et al., 2021 |
| **17** | **1,5,9-*epi*-Deoxyloganic acid*^a^*** | 4.91 | C_16_H_23_O_9_^–^ | 359.13420 | 359.12947 | 4.73 | 101.01520(15), **109.05469**(100), 113.01118(8), 135.06673(32) | ✚ | – | Aničić et al., 2021 |
| **18** | **1-*O*-Hexosyl-*epi*-deoxyloganic acid*^a^*** | 4.98 | C_22_H_33_O_14_^–^ | 521.18758 | 521.18814 | -0.56 | 135.07108(17), 153.08102(70), 191.04641(34), **197.07148**(100), 359.12834(97) | ✚ | – | Takeda et al., 1998 |
| **19** | **Nepetaracemoside B (5-Hydroxy-dehydronepetalactone hexoside) *^a^*** | 5.05 | C_16_H_23_O_8_^+^ | 343.13874 | 343.13884 | -0.1 | 135.08010(5), 163.07482(37), **181.08453**(100), 182.08778(14) | ✚ | – | Goldansaz et al., 2019 |
| **20** | **Nepetaside*^a^*** | 6.33 | C_16_H_25_O_8_^–^ | 345.15490 | 345.15159 | 3.31 | **101.01314**(100), 113.01198(77), 121.08644(23), 165.07449(26), 183.09378(23), 197.09403(14) | ✚ | – | Xie et al., 1988 |
| **21** | **Nepetariaside*^a^*** | 6.73 | C_16_H_27_O_8_^–^ | 347.17067 | 347.16632 | 4.34 | **111.07019**(100), 125.08434(9), 185.11082(7), 187.08024(11) | ✚ | – | Aničić et al., 2021 |
| ***Iridoid aglycones*** | | | | | | | | | | |
| **22** | **5-Hydroxy-dehydronepetalactone (Nepetaracemoside B aglycone)*^a^*** | 4.91 | C_10_H_13_O_3_^+^ | 181.08592 | 181.08483 | 1.09 | 105.06943(65), 107.08639(75), 117.06749(45), 127.12071(48), 135.08139(40), **163.07457**(100) | ✚ | – | Takeda et al., 1999 |
| **23** | **10-Deoxy-7,8-dihydrogenipin (7-Deoxyloganin aglycone)*^a^*** | 7.27 | C_11_H_17_O_4_^+^ | 213.11214 | 213.11051 | 1.62 | 105.06949(41), **107.08536**(100), 115.05256(10), 123.07413(11), 135.07545(30), 139.03583(32) | ✚ | – | Murai et al., 1984 |
| **24** | **Dehydronepetalactone*^a^*** | 8.62 | C_10_H_13_O_2_^+^ | 165.09160 | 165.09109 | 0.51 | 107.08362(25), 109.10033(23), 137.09555(13), **149.06192**(100) | ✚ | – | Srivastava et al., 2021 |
| **25** | **Dihydronepetalactone*^a^*** | 9.29 | C_10_H_17_O_2_^+^ | 169.12290 | 169.11996 | 2.94 | 105.06412(42), 107.08364(47), 117.00247(67), **123.11089**(100), 141.06054(98) | ✚ | – | Ali et al., 2021 |
| **26** | **Nepetalactone isomer 1*^a^*** | 10.3 | C_10_H_15_O_2_^+^ | 167.10666 | 167.10614 | 0.51 | 105.06795(19), **111.04452**(100), 119.0829(5), 121.09831(8) | ✚ | – | Karakuş et al., 2021 |
| **27** | **Nepetalactone isomer 2*^a^*** | 10.71 | C_10_H_15_O_2_^+^ | 167.10666 | 167.10697 | -0.31 | **105.06638**(100), 111.04089(18), 121.09828(19), 131.07658(10) | ✚ | – | Karakuş et al., 2021 |
| ***Other compounds*** | | | | | | | | | | |
| **28** | **Hydroxyphenyl-ethyl pentosyl-hexoside*^b^*** | 3.97 | C_19_H_27_O_11_^–^ | 431.15589 | 431.15281 | 3.07 | 101.01144(70), 101.03206(50), 113.01031(95), **119.03446**(100), 161.02996(36), 345.9348(21) | – | ✚ | Liu et al., 2014 |
| **29** | **Phenylethyl hexosyl-hexoside*^c^*** | 4.24 | C_20_H_29_O_11_^–^ | 445.17154 | 445.16719 | 4.35 | 101.01214(25), **113.01321**(100), 119.03605(29), 161.02425(6) | – | ✚ | Xu et al., 2019 |
| **30** | **Umbelliferone*^a^*** | 4.51 | C_9_H_7_O_3_^+^ | 163.03897 | 163.03854 | 0.43 | 103.05495(64), 105.06887(97), 107.04825(56), 115.05335(48), **117.03175**(100), 119.08282(76) | ✚ | – | Dienaitė et al., 2018 |
| **31** | **Schizonepetin*^a^*** | 4.64 | C_10_H_15_O_3_^+^ | 183.10210 | 183.10567 | -3.57 | 117.05798(53), **118.06487**(100), 132.07959(61), 134.09585(40), 147.10319(47), 162.05448(49) | ✚ | – | Zeren et al., 2011 |
| **32** | **Dihydromelilotoside*^b^*** | 4.78 | C_15_H_19_O_8_^–^ | 327.10854 | 327.10664 | 1.9 | **103.04338**(100), 113.01083(13), 147.03225(59), 165.04482(18) | – | ✚ | Atay et al., 2016 |
| **33** | **12-Hydroxyjasmonic acid hexoside*^a^*** | 5.28 | C_18_H_27_O_9_^–^ | 387.16606 | 387.16247 | 3.59 | 101.01384(18), 113.01294(16), **161.04984**(100), 207.09289(8) | ✚ | ✚ | Aničić et al., 2021 |
| **34** | **Hexenyl pentosyl-hexoside*^a^*** | 5.65 | C_17_H_29_O_10_^–^ | 393.17615 | 393.17130 | 4.85 | 163.04472(27), **171.09161**(100), 185.10773(50), 201.10040(34), 247.10945(72), 393.17170(73) | ✚ | ✚ | Goldansaz et al., 2019 |
| **35** | **Betonyoside*^b^*** | 6.26 | C_30_H_37_O_16_^–^ | 653.20829 | 653.20953 | -1.24 | 151.02658(16), **161.01363**(100), 179.02376(90), 459.14554(15), 621.18010(58) | – | ✚ | Li et al., 2022 |
| **36** | **1,3,3-Trimethyl-5-oxobicyclo[2.2.1]hept-2-yl acetate*^a^*** | 7.67 | C_12_H_19_O_3_^+^ | 211.13287 | 211.13065 | 2.22 | 105.06944(95), **107.08414**(100), 109.05593(44), 133.06229(39), 135.07446(61) | ✚ | – | Skorić et al., 2017 |
| **37** | **2-(2-(Hydroxymethyl)-3-methylcyclopentyl)prop-1-en-1-yl acetate*^a^*** | 11.91 | C_12_H_21_O_3_^+^ | 213.14852 | 213.14644 | 2.08 | 107.08350(28), 109.06599(23), 111.04512(51), **126.08456**(100), 135.11050(15), 198.11063(37) | ✚ | ✚ | Bottini et al., 1992 |

***t*_R_** - Mean retention time (min); Molecular formulas indicate in which ionization (**negative** or **positive**) mode a given compound is identified; *^a^*Compounds which were previously identified in *Nepeta* species; Compounds which were previously identified in some other species from Lamiaceae*^b^* family, *^c^*Compounds which were for the first time tentatively identified in genus *Nepeta*. **mDa** – Mean mass accuracy; ***NN***- *Nepeta nervosa*; ***NR*** – *Nepeta rtanjensis*; **+** stands for detected compound; **–** stands for not detected compound; **NA** – not available.

**References**

Ali, I., Ali, M., Shareef, H., Naeem, S., Khadim, A., Ali, M., Amber, F., Hussain, H., Ismail, M., Shah, S.T.A., Noor, A., & Wang, D. (2021). Phytochemical analysis and biological activities of "Cherchoomoro" (*Nepeta adenophyta* Hedge). *Journal of ethnopharmacology*, 279, 114402. <https://doi.org/10.1016/j.jep.2021.114402>

Alipieva, K.I., Kokubun, T., Taskova, R.M., Evstatieva, L.N., & Handjieva, N.V. (2007). LC–ESI-MS analysis of iridoid glucosides in *Lamium* species. *Biochemical Systematics and Ecology*, 35, 17-22. <https://doi.org/10.1016/j.bse.2006.07.004>

Aničić, N., Gašić, U., Lu, F., Ćirić, A., Ivanov, M., Jevtić, B., Dimitrijević, M., Anđelković, B., Skorić, M., Nestorović Živković, J., Mao, Y., Liu, J., Tang, C., Soković, M., Ye, Y., & Mišić, D. (2021). Antimicrobial and Immunomodulating Activities of Two Endemic *Nepeta* Species and Their Major Iridoids Isolated from Natural Sources. *Pharmaceuticals*, 14(5), 414. <https://doi.org/10.3390/ph14050414>

Atay, I., Kirmizibekmez, H., Kaiser, M., Akaydin, G., Yesilada, E., & Tasdemir, D. (2016). Evaluation of in vitro antiprotozoal activity of Ajuga laxmannii and its secondary metabolites. Pharmaceutical biology, 54(9), 1808–1814. <https://doi.org/10.3109/13880209.2015.1129542>

Bauer, J. L., Harbaum-Piayda, B., & Schwarz, K. (2012). Phenolic compounds from hydrolyzed and extracted fiber-rich by-products. *LWT*, 47(2), 246-254. <https://doi.org/10.1016/j.lwt.2012.01.012>

Bottini, A.T., Dev, V., Sah, G.C., Mathela, C.S., Melkani, A.B., Nerio, A.T., & Sturm, N.S. (1992). Cyclopentanomonoterpene enol acetates from *Nepeta leucophylla*. *Phytochemistry*, 31(5), 1653-1657. <https://doi.org/10.1016/0031-9422(92)83122-f>

Dienaitė, L., Pukalskienė, M., Matias, A.A., Pereira, C.V., Pukalskas, A., & Rimantas Venskutonis. P. (2018). Valorization of six *Nepeta* species by assessing the antioxidant potential, phytochemical composition and bioactivity of their extracts in cell cultures. *Journal of functional foods*, 45, 512-522. <https://doi.org/10.1016/j.jff.2018.04.004>

Gašić, U., Banjanac, T., Šiler, B., Božunović, J., Milutinović, M., Aničić, N., Dmitrović, S., Skorić, M., Nestorović Živković, J., Petrović, L., Todorović, M., Živković, S., Matekalo, D., Filipović, B., Lukić, T., & Mišić, D. (2023). Variation in the chemical profiles of three foxglove species in the central Balkans. *Frontiers in plant science*, 14, 1155297. <https://doi.org/10.3389/fpls.2023.1155297>

Goldansaz, S.M., Festa, C., Pagano, E., De Marino, S., Finamore, C., Parisi, O.A., Borrelli, F., Sonboli, A., & D'Auria, M.V. (2019). Phytochemical and Biological Studies of *Nepeta asterotricha* Rech. f. (Lamiaceae): Isolation of Nepetamoside. *Molecules*, 24(9), 1684. <https://doi.org/10.3390/molecules24091684>

<https://scifinder-n.cas.org/>

Karakuş, S., Atici, Ö., Köse, C., & Aydin, I. (2021). *Nepeta meyeri* essential oil ameliorates fungal infection and the antioxidant response in grapevines (*Vitis vinifera*) infections by gray mold (*Botrytis cinerea*). *Acta physiologiae plantarum*, 43, 151. <https://doi.org/10.1007/s11738-021-03320-3>

Kashchenko, N.I., & Olennikov, D.N. (2016). ХИМИЧЕСКИЙ ПРОФИЛЬ И БИОЛОГИЧЕСКАЯ АКТИВНОСТЬ ФЛАВОНОИДОВ И ФЕНИЛПРОПАНОИДОВ *NEPETA CATARIA* L. (LAMIACEAE), ИНТРОДУЦИРОВАННОГО В ВОСТОЧНОЙ СИБИРИ. *Химия растительного сырья*, (2), 25-32. <https://doi.org/10.14258/jcprm.2016021084>

Li, T., Jia, L., Du, R., Liu, C., Huang, S., Yu, H., Han, L., Chen, X., Wang, Y., & Jiang, M. (2022). Comparative investigation of aerial part and root in *Lamiophlomis rotata* using UPLC-Q-Orbitrap-MS coupled with chemometrics. *Arabian Journal of Chemistry*, 15(4), 103740. <https://doi.org/10.1016/j.arabjc.2022.103740>

Liu, Q., Hu, H.J., Li, P.F., Yang, Y.B., Wu, L.H., Chou, G.X., & Wang, Z.T. (2014). Diterpenoids and phenylethanoid glycosides from the roots of *Clerodendrum bungei* and their inhibitory effects against angiotensin converting enzyme and α-glucosidase. *Phytochemistry*, 103, 196–202. <https://doi.org/10.1016/j.phytochem.2014.02.015>

Mišić, D., Siler, B., Gašić, U., Avramov, S., Zivković, S., Nestorović Živković, J., Milutinović, M., & Tešić, Z. (2015). Simultaneous UHPLC/DAD/(+/-)HESI-MS/MS analysis of phenolic acids and nepetalactones in methanol extracts of *Nepeta* species: a possible application in chemotaxonomic studies. *Phytochemical analysis*, 26(1), 72–85. <https://doi.org/10.1002/pca.2538>

Murai, F., Tagawa, M., Damtoft, S., Jensen, S.R., & Nielsen, B.J. (1984). (1R,5R,8S,9S)-Deoxyloganic acid from *Nepeta cataria*. *Chemical & pharmaceutical bulletin*, 32(7), 2809–2814. <https://doi.org/10.1248/cpb.32.2809>

Qiao, X., Zhang, Y. T., Ye, M., Wang, B. R., Han, J., & Guo, D. A. (2009). Analysis of chemical constituents and taxonomic similarity of *Salvia* species in China using LC/MS. *Planta medica*, 75(15), 1613–1617. <https://doi.org/10.1055/s-0029-1185866>

Skorić, M., Gligorijević, N., Čavić, M., Todorović, S., Janković, R., Ristić, M., Mišić, D., & Radulović, S. (2017). Cytotoxic activity of *Nepeta rtanjensis* Diklić & Milojević essential oil and its mode of action. *Industrial Crops and Products*, 100, 163–170. <https://doi.org/10.1016/j.indcrop.2017.02.027>

Srivastava, A., Gupta, S., Singh, S., Verma, R., Srivastava, R., Gupta, A. & Lal, R. (2021). Genetic Variability and Elite Line Selection for High Essential Oil and Nepetalactone Content in Catmint (*Nepeta cataria* L.). *American Journal of Plant Sciences*, 12, 1135-1154. <https://doi.org/10.4236/ajps.2021.127079>

Sun, H., Liu, J., Zhang, A., Zhang, Y., Meng, X., Han, Y., Zhang, Y., & Wang, X. (2016). Characterization of the multiple components of *Acanthopanax Senticosus* stem by ultra high performance liquid chromatography with quadrupole time-of-flight tandem mass spectrometry. *Journal of separation science*, 39(3), 496–502. <https://doi.org/10.1002/jssc.201500915>

Takeda, Y., Kiba, Y., Masuda, T., Otsuka, H., Honda, G., Tagawa, M., Sezik, E., & Yesilada, E. (1999). Nepetaracemosides A and B, Iridoid Glucosides from *Nepeta racemosa*. *Chemical and Pharmaceutical Bulletin*, 47(10), 1433-1435. <https://doi.org/10.1248/cpb.47.1433>

Takeda, Y., Ooiso, Y., Masuda, T., Honda, G., Otsuka, H., Sezik, E., & Yesilada, E. (1998). Iridoid and eugenol glycosides from *Nepeta cadmea*. *Phytochemistry*, 49(3), 787-791. https://doi.org/10.1016/s0031-9422(98)00125-3

Xie, S., Uesato, S., Inouye, H., Fujita, T., Murai, F., Tagawa, M., & Shingu, T. (1988). Absolute structure of nepetaside, a new iridoid glucoside from *Nepeta cataria*. *Phytochemistry*, 27(2), 469-472. <https://doi.org/10.1016/0031-9422(88)83122-4>

Xu, S., Ge, X., Li, S., Guo, X., Dai, D., & Yang, T. (2019). Discrimination of Different Parts of Saffron by Metabolomic-Based Ultra-Performance Liquid Chromatography Coupled with High-Definition Mass Spectrometry. *Chemistry & biodiversity*, 16(10), e1900363. <https://doi.org/10.1002/cbdv.201900363>

Zengin G., Cvetanović A., Gašić U., Dragićević M., Stupar A., Uysal A., Şenkardes I., Sinan K.I., Picot-Allain M.C.N., Ak G., & Mahomoodally, M.F. (2020). UHPLC-LTQ OrbiTrap MS Analysis and Biological Properties of *Origanum vulgare* subsp. *Viridulum* Obtained by Different Extraction Methods. *Industrial Crops and Products*, 154, 112747. doi: <https://doi.org/10.1016/j.indcrop.2020.112747>

Zeren, L., Tong, Z., Zhang, Y., Liu, Z., Xue, P., & Xie, Y. (2011). Chemical constituents in essential oil of *Nepeta angustifolia*. *Shizhen Guoyi Guoyao*, 22(6), 1520-1521. <https://doi.org/10.3969/j.issn.1008-0805.2011.06.114>
