# Supplementary material for: Functional iridoid synthases from iridoid producing and non-producing Nepeta species (subfam. Nepetoidae, fam. Lamiaceae)
Source: Front Plant Sci. 2024 Jan 3;14:1211453. doi: 10.3389/fpls.2023.1211453 (PMC10792066; doi:10.3389/fpls.2023.1211453)
Supplement: Supplementary file 5 [file Table_4.docx]

**Supplementary Table 4.** Energy of 8-oxogeranial α,β conformations using different forcefields

| Conformation | UFF | MMFF94(s) | GAFF |
| --- | --- | --- | --- |
| 1,2 s-cis, 7,8 s-cis 8-oxogeranial | 121.104 KJ/mol | 151.535 KJ/mol | 22.0108 KJ/mol |
| 1,2 s-trans, 7,8 s-trans 8-oxogeranial | 101.209 KJ/mol | 124.955 KJ/mol | 15.3294 KJ/mol |
| 1,2 s-trans, 7,8 s-cis 8-oxogeranial | 103.674 KJ/mol | 136.007 KJ/mol | 13.5431 KJ/mol |
| 1,2 s-cis, 7,8 s-trans 8-oxogeranial | 118.639 KJ/mol | 140.185 KJ/mol | 23.1476 KJ/mol |
